# Non-muscle myosin II drives critical steps of nematocyst morphogenesis

**DOI:** 10.1016/j.isci.2023.106291

**Published:** 2023-02-28

**Authors:** Niharika Garg, Urška Knez Štibler, Björn Eismann, Moritz Mercker, Bruno Gideon Bergheim, Anna Linn, Patrizia Tuchscherer, Ulrike Engel, Stefan Redl, Anna Marciniak-Czochra, Thomas W. Holstein, Michael W. Hess, Suat Özbek

**Affiliations:** 1 University of Heidelberg, Centre for Organismal Studies, Department of Molecular Evolution and Genomics, Im Neuenheimer Feld 230, 69120 Heidelberg, Germany; 2 Institute for Applied Mathematics, Interdisciplinary Center for Scientific Computing, Heidelberg University, Im Neuenheimer Feld 205, 69120 Heidelberg, Germany; 3 Nikon Imaging Center at the University of Heidelberg, Bioquant, Heidelberg University, 69120 Heidelberg, Germany; 4 Institute of Neuroanatomy, Medical University of Innsbruck, Müllerstrasse 59, 6020 Innsbruck, Austria; 5 Institute of Zoology, University of Innsbruck, Technikerstrasse 25, 6020 Innsbruck, Austria; 6 Institute of Histology and Embryology, Medical University of Innsbruck, Müllerstrasse 59, 6020 Innsbruck, Austria

**Keywords:** Cell biology, Developmental biology

## Abstract

Nematocysts are generated by secretion of proteins into a post-Golgi compartment. They consist of a capsule that elongates into a long tube, which is coiled inside the capsule matrix and expelled during its nano-second discharge deployed for prey capture. The driving force for discharge is an extreme osmotic pressure of 150 bar. The complex processes of tube elongation and invagination under these biomechanical constraints have so far been elusive. Here, we show that a non-muscle myosin II homolog (HyNMII) is essential for nematocyst formation in *Hydra*. In early nematocysts, HyNMII assembles to a collar around the neck of the protruding tube. HyNMII then facilitates tube outgrowth by compressing it along the longitudinal axis as evidenced by inhibitor treatment and genetic knockdown. In addition, live imaging of a NOWA::NOWA-GFP transgenic line, which re-defined NOWA as a tube component facilitating invagination, allowed us to analyze the impact of HyNMII on tube maturation.

## Highlights

• A myosin II was identified as an essential factor for nematocyst morphogenesis
• Myosin II forms a compressive sheath around the growing nematocyst tube
• Blebbistatin treatment and myosin II knockdown impair nematocyst tube formation
• A NOWA-GFP transgenic line redefines NOWA as a tube antigen driving invagination

## Graphical abstract

**Figure:**
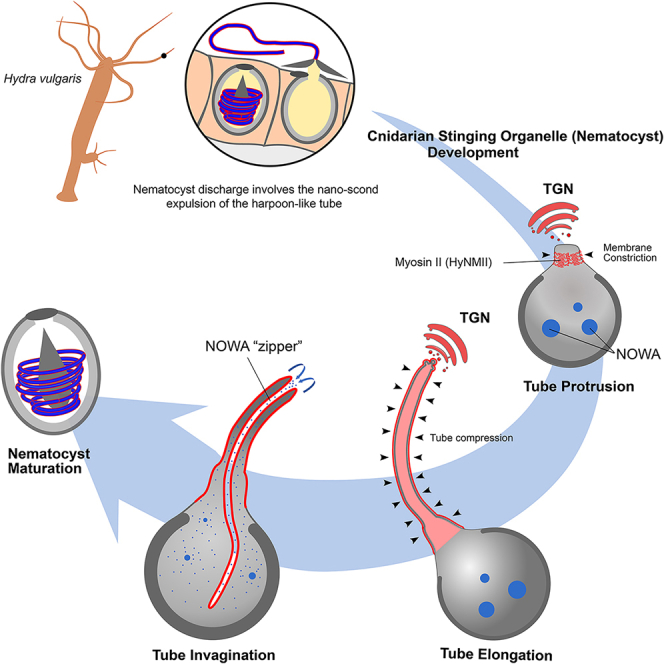

## Introduction

In eukaryotes, motor proteins of the non-muscle myosin II (NMII) family play diverse roles in the extracellular release of proteins through vesicles of the Golgi complex.^1^^,^^2^ Vesicular myosin II was originally identified as a 200-KDa phosphoprotein (p200) physically associated with the cytoplasmic side of Golgi membranes.^3^^,^^4^^,^^5^ Later studies showed that myosin II is involved in transport vesicle fission from the trans-Golgi network (TGN),^6^ a process essentially dependent on Rab6-GTPase activity.^7^^,^^8^ More recent studies propose a direct, reciprocal impact of myosin aggregation on membrane curvature.^9^^,^^10^

**Figure 1:**
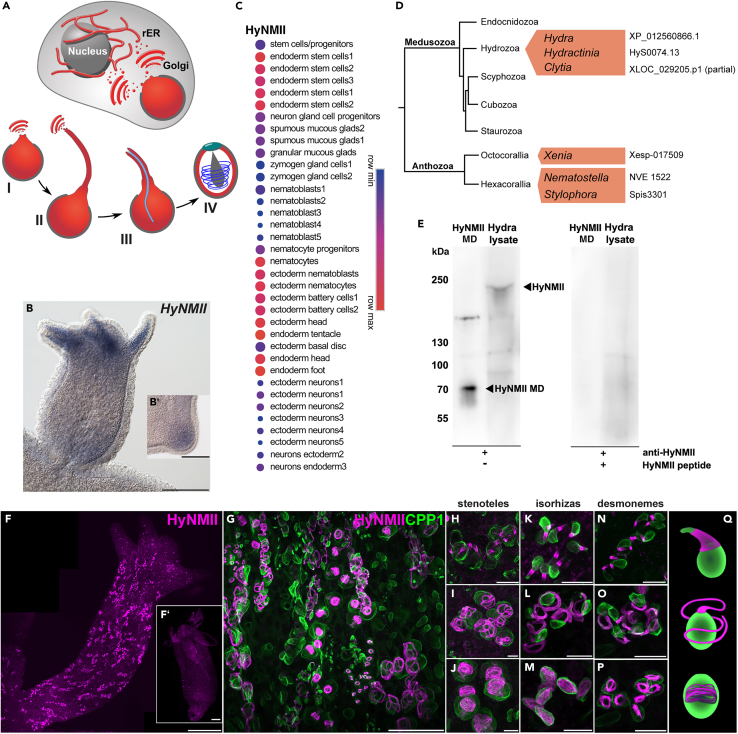
HyNMII expression pattern and immunohistochemical detection in *Hydra* whole mounts (A) Schematic drawing of nematocyst morphogenesis. The overview image of an early nematocyte shows that nematocysts are secretory products of the Golgi apparatus. Subsequent stages include tube formation, invagination, and capsule maturation. (I) Early tube protrusion. (II) External tube elongation. (III) Invagination/inversion of the tube that is later adorned with spines. (IV) Mature stenotele nematocyst with invaginated, coiled tube in the capsule matrix. rER: rough ER. (B) *HyNMII* expression is upregulated in endodermal cells of the tentacles in steady-state polyps and in early buds (B′) as determined by ISH. Scale bars = 200 μm. (C) Expression of *HyNMII* (Uniprot: T2MG36,Hydra Genome Portal 2.0 (https://research.nhgri.nih.gov/hydra/): t8308aep) in published cell clusters identified according to the presence and absence of marker genes by Siebert et al. (https://singlecell.broadinstitute.org/single_cell/study/SCP260/stem-cell-differentiation-trajectories-in-hydra-resolved-at-single-cell-resolution).^25^ Dot size and color represent the counts of mapped transcripts in the respective cell cluster. (D) Identification of cnidarian NMII homologs expressed in the nematocyte cell lineage. Published cnidarian single-cell transcriptome datasets^26^^,^^27^^,^^28^^,^^29^^,^^30^ were searched to identify non-muscular myosin genes enriched in nematocytes using *HyNMII* as a query. For species in which the nematocyte cluster was not annotated, the expression of minicollagen genes was used as a confirmation (*Xenia* minicollagens Xenia Hub (http://genome.ucsc.edu/cgi-bin/hgTracks?hubUrl=http://cmo.carnegiescience.edu/gb/hub.txt&genome=xenSp1): Xesp_000432, Xesp_001209, Xesp_001210, *Hydractinia* minicollagens: Hydractinia Genome Portal Project (https://research.nhgri.nih.gov/hydractinia): HyS0004.75, HyS0004.369, HyS0004.369, HyS0008.263). (E) Western blot detection of recombinant HyNMII motor domain and full-length HyNMII in *Hydra* lysate as indicated by arrows. The blot was performed using primary antibody solution without (left panel) or with (right panel) 1 mg/mL of the antigenic peptide used for generating the HyNMII antibody. (F–P) Detection of HyNMII by immunohistochemistry. (F) Overview of a whole *Hyda* bud stained with HyNMII antibody showing signals in developing nematocyte nests throughout the body column. Scale bar = 200 μm. F′ shows the neutralization of specific signals by pre-absorption of the primary antibody with the antigenic peptide at 1 mg/mL. Scale bar = 100 μm. (G) Double staining with HyNMII (magenta) and capsule wall marker CPP1 (green) antibodies showing nematocyte nests in different maturation stages in the gastric region. Scale bar = 50 μm. (H–P). Chronological arrangement of developing stenotele, isorhiza, and desmoneme nematocyst nests as indicated: onset of external tube formation (H, K, N), tube elongation (I, L, O), invaginated tube (J, M, P). (Q) Schematic representation of developmental stages shown in H–P. See also Figure S1 for ultrastructural detection of HyNMII in different developmental stages. Scale bars = H–P: 10 μm.

While NMII involvement has been well established for fundamental cellular processes such as cytokinesis,^11^ actomyosin networks have so far not been described to play an essential role in the generation of large membrane-encased organelles deriving from the Golgi. Here, we examine the role of a myosin II homolog from the freshwater polyp *Hydra* during nematocyst formation. Nematocysts or cnidocysts constitute one of the most complex organelles in the animal kingdom and signify the cnidarian phylum.^12^^,^^13^ While cnidarians lack a central nervous system, the mechanosensitive discharge of nematocysts represents a highly effective prey capture and defense mechanism.^14^ In *Hydra*, nematocysts are produced from specialized interstitial stem cell derivatives as a product of an intracellular secretion process.^12^^,^^15^ These nematoblasts undergo several cell divisions resulting in nest-like syncytia of 8–32 cells in the gastric region^16^ that are interconnected by cytoplasmic bridges. Each nematocyte produces a single organelle by continuous secretion of proteins into a growing post-Golgi compartment (Figure 1A).^17^^,^^18^ The mature nematocyst comprises a matrix-filled capsule of 5‒10 μm in length that runs out into a long tube. During early nematocyst morphogenesis, tube formation starts with a membrane protrusion at the apical pole of the nematocyst (Figure 1A, stage I). The tube is then elongated with its tip being surrounded by a crest of TGN vesicles and associated microtubules (Figure 1A, stage II).^17^^,^^19^ There is evidence that tube elongation is stabilized by a chondroitin matrix, which probably constitutes a significant part of the tube wall.^19^ After reaching its final length, the tube is invaginated and coiled within the capsule matrix by a hitherto unknown mechanism (Figure 1A, stage III). Final maturation of the nematocyst (Figure 1A, stage IV) involves capsule wall hardening by extensive disulfide networks of minicollagens and other structural proteins.^20^^,^^21^^,^^22^ This is accompanied by the generation of a high osmotic pressure (150 bar) through poly-gamma-glutamate synthesis and the influx of associated cations.^23^^,^^24^ After completion, the nematocytes separate and migrate into the tentacles to be incorporated into large ectodermal battery cells.

We have previously presented the proteome of isolated nematocysts from *Hydra*, which comprises more than 400 proteins.^31^ Among these, a single motor protein was identified and annotated as a non-muscle myosin II homolog. Here, we characterize the function of this myosin, designated as *Hydra* NMII (HyNMII), in the context of nematocyst morphogenesis. We show that HyNMII localizes to the cytoplasmic face of the nematocyst forming a prominent collar-like structure around the outgrowing tube and then covers the whole length of the tube during morphogenesis. Blebbistatin treatment resulted in a dramatic reduction of nematocyst nests caused by compromised tube synthesis and TGN vesicle depletion. These findings are further supported by novel antibody stainings for the nematocyst outer wall antigen (NOWA)^32^ and live imaging of a transgenic line expressing a NOWA-GFP fusion under control of the NOWA promotor. Contrasting previous findings with the H22 antibody, we show that NOWA is exclusively incorporated into the invaginating tube, a process critically hampered by BBS treatment or HyNMII depletion. Genetic knockdown of NOWA, on the other side, blocks the process of tube invagination, which is probably facilitated by a zipper-like lectin-carbohydrate interaction. Taken together, our data reveal hitherto unknown roles of the actomyosin cytoskeleton in critical steps of cnidarian nematocyst development and define NOWA as the molecular factor driving tube invagination.

## Results

### HyNMII is associated with tube morphogenesis in all nematocysts

A previous proteomic analysis of isolated nematocysts from *Hydra* revealed a single motor protein as molecular component of the mature stinging organelle.^31^ The respective gene sequence (Genbank: XM_047288384.1, Uniprot: T2MG36) was identified as a member of the subfamily of class II non-muscle myosins by BLAST and InterPro analysis and designated HyNMII. This myosin was previously shown to be expressed in endodermal cells of the tentacle base and additionally implicated to be involved in the control of cell shape changes during bud detachment.^33^ Our whole-mount *in situ* hybridization analysis confirmed this pattern, although we did not detect a consistent signal at the bud detachment site (Figure 1B). Instead, we observed an upregulation in endodermal epithelial cells during early bud formation (Figure 1B’). When we analyzed the expression signatures of *HyNMII* in the *Hydra* single-cell transcriptome database,^25^ it was found both in endodermal epithelial cell lineages, but also throughout the developmental trajectory of nematocytes with low expression in nematoblasts but high levels in nematocytes and battery cells (Figure 1C). Other interstitial stem cell derivatives like neurons or gland cells showed comparatively low expression levels. This indicated that HyNMII plays a role in nematocyst morphogenesis and, at the organismal level, could be involved in morphallactic tissue evagination processes at buds and tentacles that have been described to be induced by a curvature and reorientation of the endodermal cell layer.^34^ We also examined whether NMII homologs in other cnidarian species were enriched in the nematocyte cell lineage as well. Indeed, in all available single-cell transcriptome datasets, which include two additional hydrozoan species (*Hydractinia* and *Clytia hemisphaerica*)^26^^,^^27^ and three anthozoan species (*Xenia spec.*, *Nematostella vectensis*, and *Stylophora pistillata*),^28^^,^^29^^,^^30^ a pronounced expression of the identified *NMII* genes could be verified in the respective nematocyte cell clusters (Figure 1D), arguing for a conserved role of actomyosin networks in nematocyst development.

The molecular annotation defines HyNMII as a ∼224-kDa myosin heavy chain protein comprising an N-terminal SH3 domain followed by the consensus motor domain, a light-chain binding region with a single IQ motif, and the coiled-coil tail region that induces dimerization. The primary sequence of HyNMII lacks a signal peptide motif at the protein’s N-terminus, arguing for a cytoplasmic localization of the mature protein. To localize HyNMII in *Hydra*, we generated a polyclonal antibody against an epitope in the head domain with low sequence similarity to other myosin proteins identified in the *Hydra* genome (LSQLYKEQLQGLMNTL). The HyNMII antibody recognized a high molecular weight band (∼230 kDa) in Western blots of whole *Hydra* lysates, which corresponds well with the calculated molecular mass of 224,4 kDa (Figure 1E, left). The antibody also recognized the recombinant motor domain protein expressed in *E. coli*, which has a predicted molecular mass of 80,3 kDa, but migrates at 72 kDa. Both signals were lost by pre-absorption of the primary antibody with the antigenic peptide used for immunization (Figure 1E, right).

In whole-mount immunostainings, HyNMII was exclusively detected in nests of differentiating nematocytes throughout the body column (Figure 1F). This pattern is reminiscent of diverse other nematocyst-specific factors whose detection is restricted to the morphogenetic stages in the gastric region, like minicollagens^22^^,^^35^ or Cnidoin.^20^ Antibody staining in mature nematocysts in the tentacles is often hampered by the dense protein polymer of the capsule wall structure. In control stainings where we preabsorbed the primary antibody with the antigenic peptide, the signal was markedly reduced (Figure 1F′). To confirm that the HyNMII staining is associated with developing nematocyst nests, we performed co-stainings with anti-CPP1 antibody (Figures 1G–1P). The cnidarian proline-rich protein 1 (CPP1) is a structural component of the capsule wall and the respective antibody marks the developing capsule body without tube.^36^ In close-up views of the gastric region, HyNMII was detected in tubes of all capsule types throughout different developmental stages (Figure 1G). When we sorted nests of the stenotele, isorhiza, and desmoneme capsule types by selecting three characteristic stages (I, onset of external tube protrusion; II, tube elongation; IV, maturing capsule with invaginated tube, see also Figures 1A, 4A, S1A, and S1B), HyNMII initially forms a prominent collar-like structure around the tube base (Figures 1H, 1K, 1N, and 1Q upper schematic). During elongation, HyNMII covers the whole length of the tube (Figures 1I, 1L, 1O, and 1Q middle schematic) and could also be detected along tubes coiled in the capsule matrix after invagination (Figures 1J, 1M, 1P, and 1Q lower schematic). This signal was lost in fully mature nematocysts in battery cells of the tentacles (Figure 1F). Attachment of HyNMII to tubes at the three main stages of morphogenesis was also evaluated by immunoelectron microscopy (Figures S1B–S1E). The immunogold label was observed at the cytoplasmic face of the membrane of forming (Figure S1C) or elongated (Figure S1D) external tubes, as well as at the electron-lucent middle layer of the invaginated tube (Figure S1E), which is in accordance with our immunofluorescence staining results. In summary, HyNMII is associated with critical steps of tube formation during nematocyst morphogenesis in *Hydra*, beginning with a prominent collar-like sheath around the tube protruding apically from the young capsule. In epithelial cells, distinct HyNMII staining patterns were not recognizable, as would have been expected from the gene expression profiles. We presume that this is due to the strong enrichment of HyNMII at the nematocyst tube structure and its probably more diffuse distribution in epithelial cells.

### Blebbistatin treatment inhibits nematocyst tube morphogenesis

**Figure 2:**
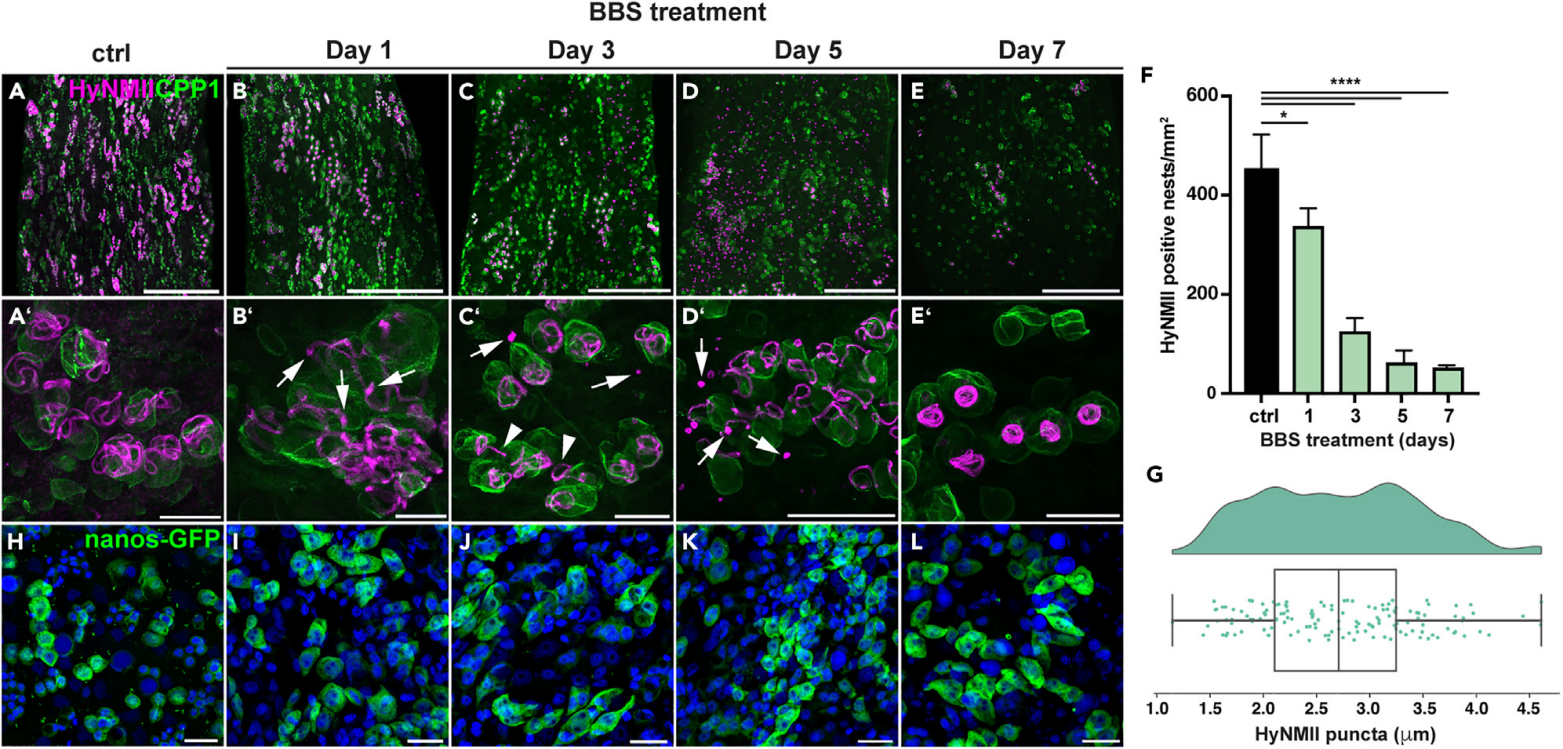
Blebbistatin treatment leads to tube disintegration (A–E) Depletion of HyNMII-positive nests in the gastric region of polyps that were continuously treated with BBS, fixed and stained for HyNMII (magenta) and CPP1 (green) at different time points as indicated (see also Figures S2A–S2D, which shows the consecutive depletion of nematocyst morphogenetic stages during BBS treatment). Note the appearance of large HyNMII-positive puncta that increase in number from day 1–5 (B–D). (A′–E′) High-magnification images of nematocyst nests in the gastric region of BBS-treated polyps demonstrate HyNMII dissociation from tubes in the form of dot-like structures. Images in A′–D′ show stenoteles in the stage of tube elongation as in Figure 1I and corresponding to stage II in Figure 1A. E′ shows a stenotele nest with coiled internal tubes as in Figure 1J and corresponding to stage IV in Figure 1A. Arrows in B′ indicate bulges mostly forming at tube tips. Arrows in C′ indicate HyNMII-positive dots, while arrowheads indicate external parts of partly invaginated tubes. Arrows in D′ indicate HyNMII-positive puncta in close vicinity to external tubes. See also Figures S2E–S2H, which summarizes typical tube-associated phenotypes observed upon BBS treatment and S2I-J for localization of HyNMII puncta in tentacles. (F) Quantification of HyNMII-positive nests in the gastric region of BBS-treated polyps (see also Table S1). Data represent mean ± SD from 3 animals compared to control polyps. ∗∗∗∗p value <0.0001, ∗p value <0.05. The data were analyzed using a one-way ANOVA test. (G) Rain cloud plot showing the size distribution of HyNMII-positive dots in BBS-treated animals (see also Table S2). The dots were measured along their longest diameter in the upper body region of 3 different animals at day 5 of BBS treatment. (H–L) Polyps of the Cnnos1::GFP transgenic line continuously treated with BBS for 7 days as in A–E show no significant reduction in GFP-positive i-cells in the gastric region. See Figures S2K and S2L for the effect of longer treatments. Cell nuclei were stained with DAPI. Scale bars = A–E: 100 μm; A′–E’: 25 μm; H–L: 20 μm.

Nematocytes derive from the continuously dividing i-cell population in *Hydra’s* body column. i-cells have to proliferate for 2–4 days to produce nematocyte nests, followed by the differentiation phase of the capsule of 2.5–3.5 days.^37^ The turnover time for mature nematocytes in the battery cells of the tentacles has been determined to be between 7 and 9 days.^38^ We assumed therefore that a continuous treatment with low, not cytotoxic concentrations of the myosin II inhibitor blebbistatin (BBS) for 7 days should effectively deplete most of the nematocyst developmental stages. We monitored the effect on nematocyst morphogenesis by a HyNMII/CPP1 co-staining to be able to distinguish between tube and capsule body phenotypes. As shown in the overview images in Figures 2A–2E, HyNMII-positive developmental stages continuously decreased with BBS treatment and almost completely disappeared by day 7 (Figures 2E and 2F). Interestingly, this depletion affected stage I–III nematocysts disproportionally as compared to stage IV nematocysts; the latter were unaffected during the first 2 days of treatment and increased from 19% to 43% of the total nematocyst population at day 7 (Figures S2A–S2D), indicating that late-stage nematocysts with invaginated tubes mostly escaped the inhibitory effect of the drug. BBS treatment was accompanied by the appearance of large HyNMII-positive puncta throughout the gastric region, which were most abundant at day 5 of the treatment (Figure 2D). High-magnification images revealed that these punctate elements have a considerable size with an average diameter of 2.7 μm (Figure 2G) and are mostly associated with nematocyst nests exhibiting external tubes (Figures 2B′–2D′). Indeed, such morphogenetic stages showed prominent bulges at the tube tips and sometimes along the whole length of the tube which were not present in controls (Figures 2A′–2B′, S2E, and S2F). At days 3–5 of BBS treatment, HyNMII-positive puncta could be regularly observed in close vicinity to nematocyst nests with external tubes, indicating their detachment from developing tubes (Figures 2C′–2D′ and S2F). Tube lengths marked by HyNMII staining, in particular those of stenoteles, appeared significantly reduced (see also Figure S2G) or tubes were completely missing (Figure S2H) when compared to untreated controls (Figures 2A′ and S2E). In some cases, tube invagination seemed to be hampered as evidenced by nests, where only a fraction of nematocysts showed invaginating tubes (Figure 2C′), a transient morphogenetic stage that normally is strictly synchronized within a syncytium. Impaired tube development by BBS treatment was apparently associated with disintegration of the whole nematocyst, suggesting that capsules with malformed tubes do not complete their maturation process (Figure S2G). After 7 days of BBS treatment, the remaining HyNMII-positive developmental stages were to a disproportionally large fraction such with coiled internal tubes (Figures 2E, 2E′, S2C, and S2D). Apart from this, the body column of the animal contained mostly single mature nematocysts as also evidenced by the CPP1 staining (Figure 2E), suggesting that these nematocysts had passed BBS-sensitive stages (tube protrusion and elongation) before onset of BBS treatment.

Interestingly, HyNMII-positive puncta were also detected in the tentacle region of BBS-treated polyps (Figures S2I and S2J), which suggests that nematocytes harboring disintegrated nematocysts still migrate toward the tentacles. Possibly, the remnants of malformed capsules are phagocytosed by the epithelial cells into which migrating nematocytes are integrated. To exclude that the depletion of nematocyte nests by BBS treatment is a consequence of inhibited i-cell proliferation, we made use of a transgenic line expressing GFP under control of the *Hydra nanos* promotor (Cnnos1::GFP).^39^ In untreated, steady-state polyps of this strain, the gastric region is densely populated with a constant number of GFP-positive cells reflecting well-balanced i-cell proliferation and differentiation processes (Figures 2H and S2K). BBS treatment for 7 days did not diminish GFP-positive i-cells in these animals, indicating that the proliferation of i-cells is less sensitive to BBS compared to nematocyst morphogenesis (Figures 2H–2L), which might be due to the high mechanical strain of the nematocyst’s tube structure. Only when BBS treatment was extended to 14 days, the i-cell population was largely depleted as well (Figure S2L), probably caused by an additive inhibitory effect on diverse myosin-dependent cellular processes. We conclude from these results that the progression of nematocyst morphogenesis is essentially dependent on HyNMII activity and BBS treatment leads to a depletion of developmental stages by destabilizing external tube structures.

### TGN vesicles at growing nematocysts are rapidly depleted by blebbistatin

**Figure 3:**
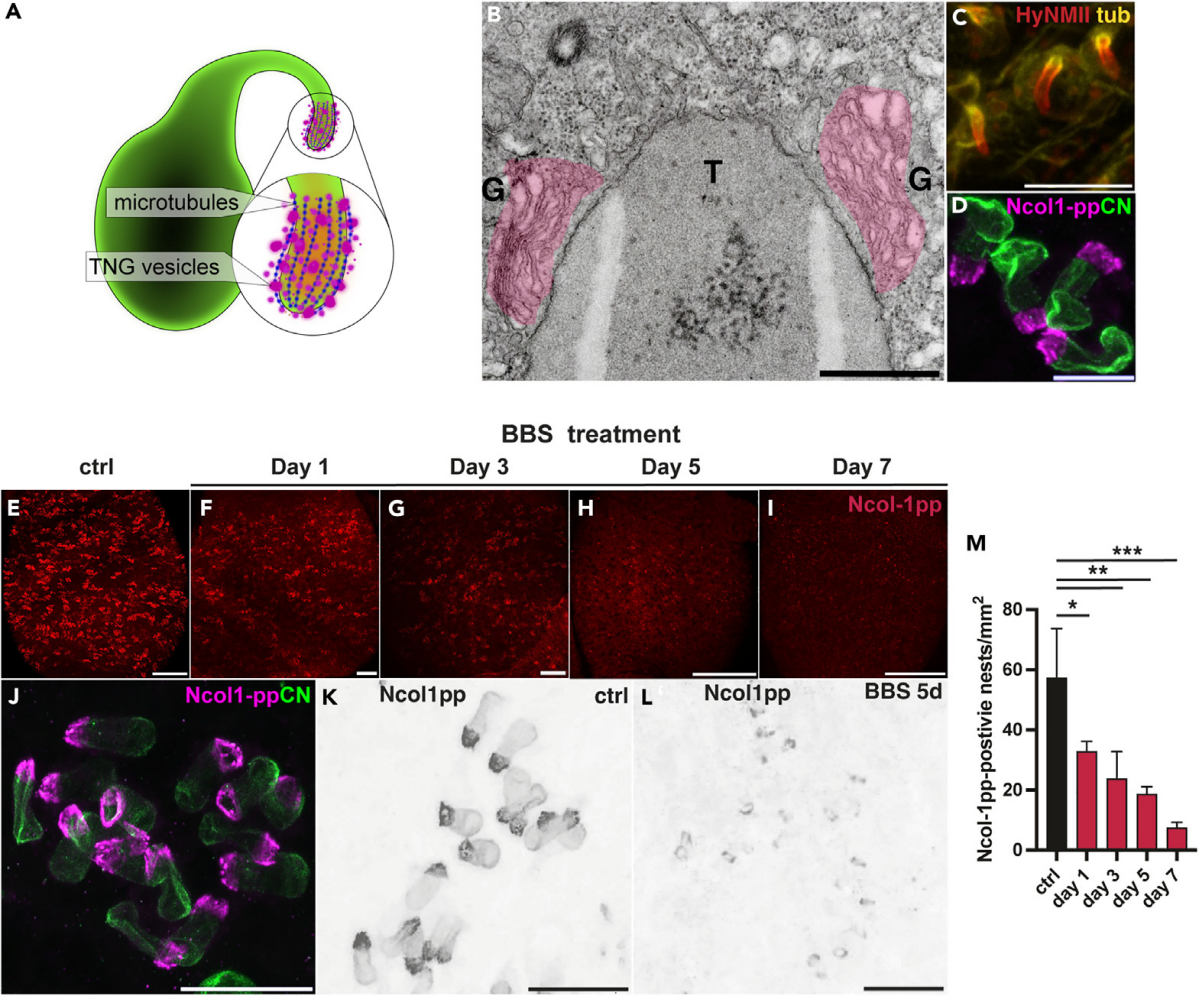
Blebbistatin depletes TGN vesicles at growing nematocyst (A) Schematic drawing of early nematocyst development showing the arrangement of microtubules and TGN vesicles around the tip of the growing tube. (B) Electron micrograph of a section showing the nematocyst tube (T) tip surrounded by stacks of the Golgi apparatus (G, pseudocolorized). (C) Double staining with HyNMII (red) and beta-tubulin antibodies (yellow) shows a crest of microtubules surrounding the external tube tips of growing nematocysts. (D) Double staining with Ncol-1pp (magenta) and the capsule wall marker Cnidoin (CN, green) marks the crown of TGN vesicles at the apical protrusion of the growing nematocyst. Scale bars = 10 μm. (E–I) BBS treatment leads to a rapid depletion of Ncol-1pp-positive TGN vesicles in the body region. Days of treatment are indicated. Scale bars = E, F, H, I: 200 μm; H: 100 μm. (J) Nematocyst nest in the gastric region stained with Ncol-1pp (magenta) and Cnidoin (CN, green). Scale bar = 20 μm. (K and L) High-magnification images from animals in E and H showing depletion of TGN vesicles in a single nematocyst nest. For clarity, the Ncol-1pp-stained images (magenta) were converted to grayscale and shown in inverted contrast. Scale bars = 20 μm. (M) Quantification of Ncol-1pp-positive nests in the gastric region of BBS-treated polyps (see also Table S3). Data represent mean ± S.D. from 3 animals compared to control polyps. ∗∗∗p value <0.0005, ∗∗p value <0.005. ∗p value <0.05. The data were analyzed using a one-way ANOVA test.

The fact that BBS treatment led to a complete depletion of nests including early stages preceding tube formation suggested a possible role of myosin II already in the generation of the initial, TGN-derived nematocyst vesicle. We investigated this hypothesis by choosing a molecular marker for TGN vesicles targeted for the nematocyst. During its morphogenesis, the apical pole of the growing nematocyst and the tip of the tube are surrounded by a cluster of TGN vesicles that continuously fuse with the nematocyst membrane^17^ (Figures 3A and 3B). These transport vesicles are organized by the microtubule basket that surrounds the external tube and carry structural proteins that will shape the capsule (Figure 3C). We have previously shown that an antibody directed against the minicollagen-1 (Ncol-1) pro-peptide (pp) sequence, which is cleaved before the mature Ncol-1 protein is secreted into the nematocyst, visualizes the crest of TGN vesicles surrounding the growing tube^19^ (Figure 3D). Ncol-1 is a major constituent of the nematocyst and gets incorporated into the capsule wall structure during its final maturation stage.^35^

In BBS-treated animals, we observed a significant depletion of Ncol-1pp-positive nematocyst nests already after 1 day (Figures 3E, 3F, and 3M), followed by a continuous loss during the following days of treatment (Figures 3G–3I and 3M). In addition, Ncol-1pp-positive vesicles at individual nematocysts (Figure 3J) were markedly reduced in number during BBS treatment (Figures 3K and 3L). This indicates that HyNMII, in addition to its role in tube formation, is essentially involved in the generation of cargo vesicles from the Golgi complex targeting the growing nematocyst, in agreement with findings that myosin II plays a role in the production of transport vesicles from the TGN.^6^ We presume that BBS treatment effectively inhibits the generation of new nematocysts in nematoblasts and that the reduction of cargo vesicles contributes to defects in capsule and tube synthesis due to a reduced supply of structural proteins. It has to be stressed, though, that the above shown defects in tube elongation by BBS (Figure 2) are likely a direct result of HyNMII inhibition at the tube’s surface, as we did not observe comparable defects of the capsule wall integrity, which relies on structural protein supply from the TGN as well.

### NOWA is re-defined as a late tube antigen that facilitates invagination

NOWA, a C-type lectin with multiple minicollagen cysteine-rich domains at its C-terminus, was originally described as a component of the capsule wall.^32^ The monoclonal antibody H22 introduced in that study as being specific for NOWA was originally generated using a preparation of isolated nematocysts, and stained the outer wall of mature capsules.^40^ The H22 antigen was later isolated by subjecting nematocysts to 2D electrophoresis and Edman degradation of a protein spot correlating with H22 Western analysis, which finally resulted in the identification of the NOWA protein sequence. Further analysis revealed that H22 antibody recognition depended on N-linked glycosylation of its antigen.^32^ A polyclonal antibody raised against the C-type lectin domain (CTLD) of NOWA recognized a protein band migrating at 88 kDa, which matched the molecular mass of the antigen detected by the H22 antibody.^32^ It was therefore concluded that the antigens of the H22 and CTLD antibodies are identical.

**Figure 4:**
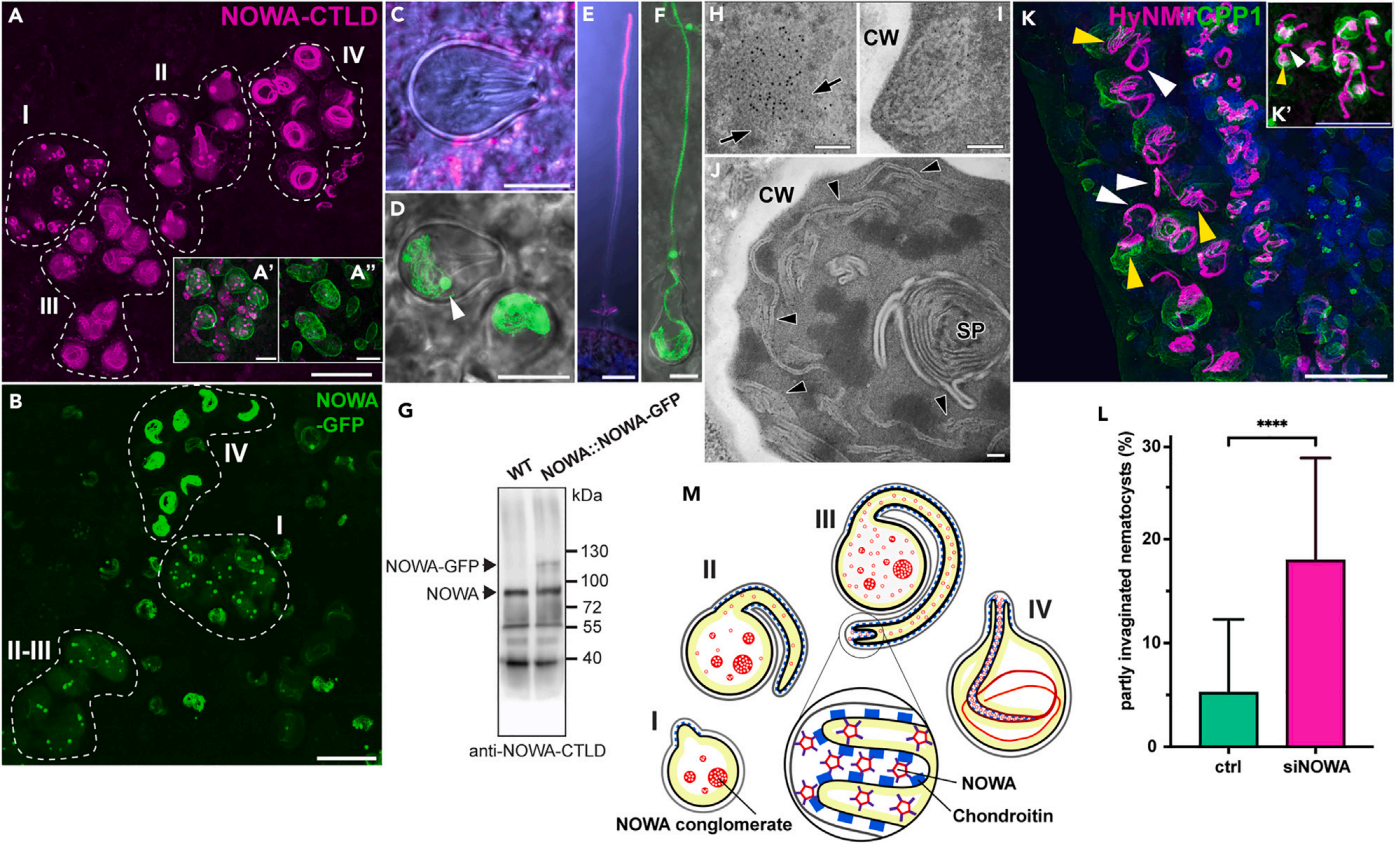
NOWA is a late tube component that facilitates invagination (A) Detail of the gastric region illustrating major developmental stages of stenoteles stained with NOWA-CTLD antibody. I, onset of external tube formation with several large NOWA granules inside the nematocyst capsule. II; tube elongation at the beginning of, or close to invagination. III, late invagination stage showing the helical shaft region and the partly coiled tube. NOWA spots are completely dissociated throughout the capsule matrix. IV, mature capsule characterized by condensation of NOWA at the fully coiled tube structure and a virtually clear capsule lumen. The inserts show the reduction of NOWA-CTLD-stained structures in animals electroporated with NOWA siRNAs as compared to siGFP-treated controls. (A′) Stenotele nest at stage I stained with NOWA-CTLD (magenta) and CPP1 (green) antibodies. (A″) Stenotele nest as in A′ in an animal treated with NOWA siRNAs showing a reduction of NOWA aggregates in the capsule matrix. (B) Detail of the gastric region illustrating stenotele developmental stages in NOWA::NOWA-GFP transgenic animals shows similar pattern as in A. (C) In mature capsule of the tentacles, the NOWA-CTLD antibody does not stain the tube within the capsule due to the impermeable capsule wall. (D) In contrast to C, mature capsules in transgenic animals show NOWA-positive internal tubes. The arrowhead marks a NOWA-GFP protein aggregate, which normally is dissolved in mature capsules of wild-type animals. See also Figure S3 and Video S1 for visualization of NOWA protein dispersal during invagination. (E) Tube of discharged nematocyst stained with NOWA-CTLD antibody. (F) NOWA-GFP marks both the internal and external tube in a partly discharged mature nematocyst. Scale bars = A–B: 25 μm; A′–A″, C–F: 5 μm. (G) In a Western blot using isolated nematocysts from wild-type and transgenic NOWA::NOWA-GFP animals, NOWA is detected by the NOWA-CTLD antibody at the expected molecular mass of 88 kDa. A weaker second band at about 115 kDa in the transgenic capsule sample marks the NOWA-GFP fusion protein. (H–J) NOWA-CTLD detection by cryosection immunogold-EM (formaldehyde fixation). (H) In stage I nematocysts (see Figures 1A, 1H, and 4A) with protruding tubes, NOWA-CTLD was confined to 0.5–1 μm wide spots within the capsule which first comprised honeycomb-like patterns (arrows) and more loosely arranged strand-like elements in stage II nematocysts (I). Stage III nematocysts showed NOWA-CTLD along the profiles of the inverted tube (J, arrowheads). The multi-layered structure in the center is the spines (SP) that differentiate within the lumen of the tube shaft region. CW = capsule wall. Scale bars = 200 nm. (K) Double staining with HyNMII (magenta) and CPP1 (green) antibodies of a stenotele (nest on the left side), and isorhiza (K′) nest showing thickened double-layered external tubes (white arrowheads) and fine threads of invaginated tubes in the capsule matrix (yellow arrowheads) in siNOWA-treated animals. Scale bars = K, K’: 25 μm. (L) Percentage of nematocyst nests showing partially invaginated tubes in siNOWA and control electroporated *Hydras* (see also Table S4). Data represent mean ± SD from 20 (ctrl) and 34 (siNOWA) random areas of 85,434.286 *μ*m^2^ in the gastric regions of 3 animals in each treatment group. ∗∗∗∗p value <0.0001. The data were analyzed using an unpaired t-test. (M) Scheme showing the proposed “zipper” mechanism for tube invagination facilitated by NOWA. NOWA conglomerates that appear as large droplet-like structures in the capsule matrix of stages I and II dissociate during the invagination phase (III–IV) into smaller particles connected by cysteine links. We assume that these pass the tubule wall to interact with the chondroitin matrix on the outer surface of the tube via NOWA’s lectin domains. This process could cross-link the invaginating tube surfaces driving invagination in the form of a carbohydrate-lectin “zipper”.

When we tested the CTLD antibody in whole-mount immunofluorescence stainings, the observed pattern did not match the one described for the H22 antibody. As shown in Figure 4A, the CTLD antibody predominantly stained coiled, fully invaginated tubes in late-stage nematocyte nests (stage IV). Early developmental stages (stages I–II, tube protrusion and elongation) showed large NOWA-CTLD-positive spots in the capsule matrix very similar to those observed for Ncol-15 and spinalin.^41^ During invagination (stage III), these spots disappeared and the helical shaft region as well as the loosely arranged invaginated tube were stained. In fully mature nematocysts of the tentacles, undischarged tubes were not stained due to the impermeable capsule wall polymer (Figure 4C), but discharged nematocysts showed a staining along the whole tube (Figure 4E).

The specificity of the NOWA-CTLD antibody was verified by silencing *NOWA* gene expression (see below), which led to markedly reduced NOWA protein aggregates in stage I nematocyst nests (Figures 4A’ and 4A″). To further confirm the unexpected staining pattern, we generated a transgenic line expressing a NOWA-GFP fusion protein under control of the NOWA promotor (NOWA::NOWA-GFP). Developing nematocysts in this strain showed an identical pattern as in anti-NOWA-CTLD-stained wild-type specimens (Figure 4B), while in mature capsules of transgenic animals the GFP signal was visible in both discharged and undischarged nematocysts (Figures 4D and 4F). Interestingly, protein aggregates formed by NOWA-GFP were only partially dissolved in transgenic mature capsules, indicating that the N-terminal GFP fusion might interfere with this process (Figures 4D and 4F). The presence of the tagged NOWA protein in the transgenic line was confirmed by Western blot using isolated nematocysts from wild-type and transgenic animals, which showed an additional band at about 115 kDa corresponding to the molecular mass of the fusion protein (Figure 4G). The NOWA::NOWA-GFP transgenic animals allowed us to monitor tube invagination and maturation by time-lapse imaging (Video S1). The movie demonstrates that tube invagination is closely correlated with the dispersal of the NOWA aggregates and the gradual condensation of NOWA protein at the coiled tube structure within the capsule. Apparently, the droplet-like NOWA structures serve as deposits that undergo a phase conversion during tube invagination, as described for a wide array of proteins,^42^ and are then fully consumed while being integrated into the tube. This process takes about 6–7 h (Video S1, Figure S3).

Video S1. Time lapse of nematocyte nest in body column of NOWA::NOWA-GFP animals with corresponding bright field (one cell of the nest is highlighted with white circle), related to Figure 4 At time 0, invagination is in process and NOWA-GFP is weakly associated with the external tube (white arrow) and found in the capsule matrix as well as bright aggregates in the nematocyte cytoplasm. In the following 3 h, NOWA relocalizes from aggregates in the capsule matrix to the invaginated tubule highlighted by gray arrow. Around 7 h after onset of imaging, internalization of the tube is completed, as indicated by the start of formation of the stylet apparatus visible in bright field (black arrow) and a change in capsule shape (highlighted in Figure S3). Scale bar = 20 μm.

Finally, we localized NOWA at the ultrastructural level by immunoelectron microscopy (immuno-EM) (Figures 4H–4J). About (0.2-)0.5–1 μm wide, well-defined spots with strong NOWA-CTLD immunogold label were first recognized in the matrix of nematocysts with early-stage external tubes. The spots consisted of tangled lattice-like elements that locally displayed regular honeycomb patterns with quite constant mean “cell”-diameters of approximately 13 nm (Figure 4H). Subsequently, this configuration changed and the individual fibers appeared more loosely arranged, measuring about 24 nm in diameter (Figure 4I). Eventually, NOWA was located along the profiles of the inverted tube (Figure 4J).

In summary, our results clearly demonstrate that the target of the CTLD antibody, designated as NOWA, is a major component of the tube and different from the H22 antigen. We assume that the protein spots of NOWA and the unknown H22 antigen overlap in 2D gels of nematocyst preparations and that the more abundant NOWA protein had been identified by the original Edman analysis leading to its initial description as the H22 antigen.

We also examined the possible role of NOWA for tube morphogenesis by electroporating animals with two NOWA-specific siRNAs targeting unique sequences coding for the protein’s C-terminus. Interestingly, by using the HyNMII antibody, we observed a notable enrichment of early tube invagination stages, which normally are rare events and, due to their transient nature, difficult to capture. This stage is characterized by a thickened, double-layered external tube and, emerging from this, a more delicate thread of the invaginating distal tube that is partly coiled in the capsule lumen. Figure 4K shows nests of stenotele and isorhiza (Figure 4K’) type nematocysts with partly invaginated tubes in NOWA siRNA-treated polyps. A quantification of nematocyst nests captured in the state of early invagination showed an increase to an average of 18% in animals electroporated with NOWA siRNAs compared to 5.2% in control animals (Figure 4L). These findings indicate that NOWA might be the molecular factor driving tube invagination, probably by interacting with the tube’s chondroitin matrix^19^ via its lectin domain (Figure 4M).

### NOWA reveals compromised tube formation in animals treated with BBS or HyNMII siRNAs

**Figure 5:**
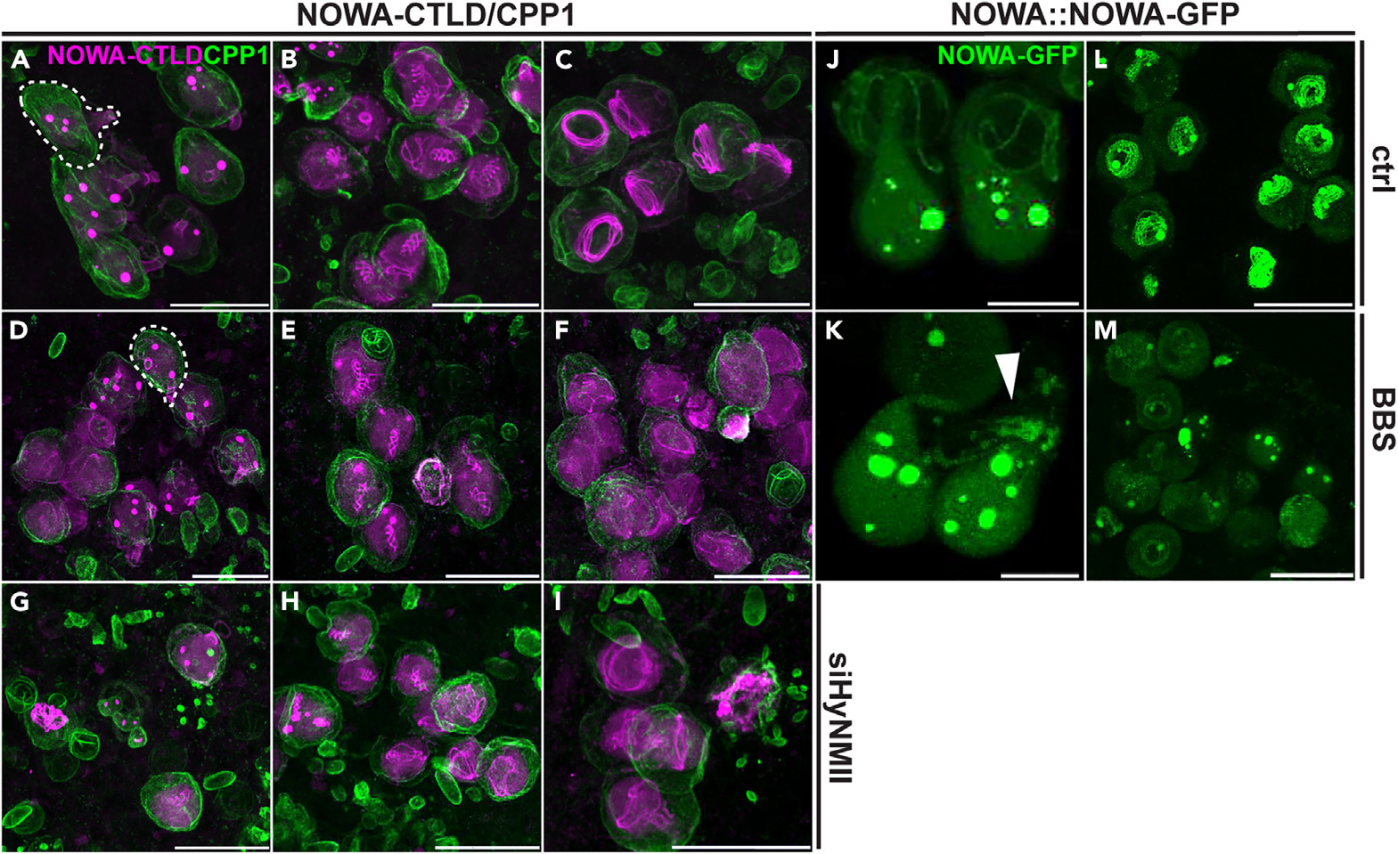
NOWA indicates compromised tube formation in BBS- and siHyNMII-treated animals (A–F) Double staining of control and BBS-treated (4 days) wild-type *Hydras* with NOWA-CTLD and CPP1 antibodies. (A and D) Early stage of nematocyst development with tube protrusion (see also stage I, Figure 4A) at the apical nematocyst pole (dotted line) and large NOWA-positive protein spots showing asynchronous development in BBS-treated animal. (B and E) Intermediate stages (see also stage III, Figure 4A) with beginning incorporation of NOWA into the triple helical folds of the shaft structures and tubes after invagination show irregular shafts and missing tubes in BBS-treated animal. Note that the NOWA protein spots are only partly dissolved in (E). (C and F) Late-stage nematocysts before final maturation (see also stage IV, Figure 4A) lack the coiled NOWA-positive tube structures (C) in BBS-treated animals, in which the NOWA protein remains as an amorphous aggregate (F). (G–I) NOWA-CTLD and CPP1 double staining of nematocyst nests in animals electroporated with *HyNMII* siRNAs. (G) Stage I showing partial loss and asynchronous development of nematocysts. (H) Stage III showing compromised and asynchronous tube formation after invagination. (I) Incomplete tubes and partly disaggregated capsule in stage IV nematocyst nest. See also Figure S4 for more detailed phenotypes observed after *HyNMII* knockdown. (J) Stage II nematocysts in untreated NOWA::NOWA-GFP polyps visualized by live imaging show NOWA protein aggregates in the capsule matrix and intact elongated external tubes. (K) In BBS-treated *Hydras* (5 days), stage II nematocysts show disaggregation of the tube structure into vesicular bodies (white arrowhead) similar to the HyNMII-positive puncta observed in Figure 2. (L) Mature nematocysts (stage IV) in untreated NOWA::NOWA-GFP *Hydras* are marked by GFP-positive coiled tubes. (M) In BBS-treated animals (5 days), late-stage nematocysts exhibit failed tube maturation as evidenced by a lack of GFP-positive tube structures. Scale bars = A–L, L–M: 20 μm. J–K: 10 μm.

NOWA’s unexpected role as a late tube component allowed us to analyze the effect of BBS treatment on the maturation of the invaginated tube. To visualize both, the capsule wall and tube, we performed a NOWA-CTLD/CPP1 double staining in animals treated with BBS up to five days. Besides an overall depletion of nematocyst nests as observed before (Figures 2 and 3), we noticed phenotypes indicative of a compromised tube formation and nematocyst maturation. Early developmental stages of stenoteles exhibiting an outward protrusion of the tube shaft and several large NOWA-positive spots inside the capsule matrix (see also stage I, Figure 4A) showed incomplete nematocyst nests and an asynchronous development within individual nests as compared to controls (Figures 5A and 5D). Intermediate stages (see also stage III, Figure 4A) in control animals were characterized by a dispersion of NOWA aggregates in the capsule matrix followed by a gradual condensation at the triple helical folds of the shaft and at the invaginated tube (Figure 5B). In BBS-treated *Hydras*, these stages contained malformed shaft structures associated with only partly dissociated NOWA spots and missing tubes (Figure 5E). Late stages that normally show NOWA-positive coiled internal tubes cleared of dispersed NOWA protein (Figure 5C, see also stage IV, Figure 4A) were increasingly substituted by nests lacking any recognizable tube structures and characterized by a diffuse distribution of NOWA protein within the capsule matrix (Figure 5F).

We attempted to verify these findings by electroporating polyps with *HyNMII*-specific siRNAs, which led to a significant reduction of the HyNMII signal compared to siGFP-treated controls (Figures S4A–S4F). In siHyNMII-treated animals, we detected comparable phenotypes of asynchronous development and tube malformation as obtained by BBS treatment (Figures 5G–5I and S4G–S4L)). In addition, nests were often incomplete (Figures 5G and S4G–S4L) and showed disintegration of individual nematocysts (Figures 5I and S4J). We concluded from these results that due to a compromised tube formation by BBS treatment as also evidenced by HyNMII staining (Figure 2), tube invagination failed or was incomplete preventing NOWA from finding its target interaction partner on the fully developed tube surface. This assumption was confirmed by NOWA::NOWA-GFP transgenic animals treated with BBS, in which stage II nematocysts showed disintegration of the external tubes into vesicular structures (Figures 5J and 5K), confirming that the observed HyNMII-positive puncta (Figure 2) not only contain dissociated HyNMII protein aggregates but also tube material. In addition, nests of mature nematocysts, which normally are GFP-positive only in their coiled internal tubes (Figure 5L), were mostly displaced by nematocyst “ghosts” that apparently had failed to undergo normal tube development (Figure 5M).

### Cytochalasin D disrupts external tube stability

As described previously and also shown above (Figures 3A and 3C), the nematocyst tube is stabilized by an array of microtubules surrounding its distal end.^17^^,^^32^ So far, an interaction of the actin cytoskeleton with the developing nematocyst has not been described. Although we have not been able to visualize actin configurations that correlate with the prominent staining of HyNMII around the external tube by phalloidin staining, electron microscopy yielded first, though not exhaustive information on the presence and localization of actin in developing nematocytes. Two different, complementary immuno-EM approaches gave consistent results, but each had some constraints: Pre-embedding labeling yielded adequate label intensity, but moderate ultrastructure preservation (Figures S5A–S5C) because of the strong detergent extraction; post-embedding labeling of cryosections resulted in good ultrastructural preservation, but comparatively weak actin signal in nematocytes—in contrast to all other *Hydra* tissues (i.e., muscle, nerve, and subapical networks in epidermal/epithelial and gastric cells; data not shown). In particular, actin labeling occurred throughout the nematocytes’ cytoplasm (Figures S5A–S5C), sometimes in loose clouds (as seen in permeabilized polyps), or locally associated with organelles, such as Golgi/TGN (as seen in cryosections). Enrichment of immunogold particles was further visible in the vicinity of elongating tubes (e.g., Figure S5B). They were not directly attached to the membrane surrounding the tube, but some of them appeared to co-localize with fine, filamentous elements (Figure S5C). In line with this, single microfilaments or loose meshworks with appropriate filament diameters of 6–7 nm could be recognized in polyp samples processed by means of rapid cryofixation (e.g., Figure S5D) or optimized chemical fixation during entire nematocyte morphogenesis. Only older nematocytes with inverted tube regularly showed bundled microfilaments similar to vertebrate stress fibers, with filament diameters in the range of 7 nm (Figure S5E) and being partly actin-positive (data not shown). Thus, comprehensive insight into the actin architecture during nematocyte development requires further analyses.

**Figure 6:**
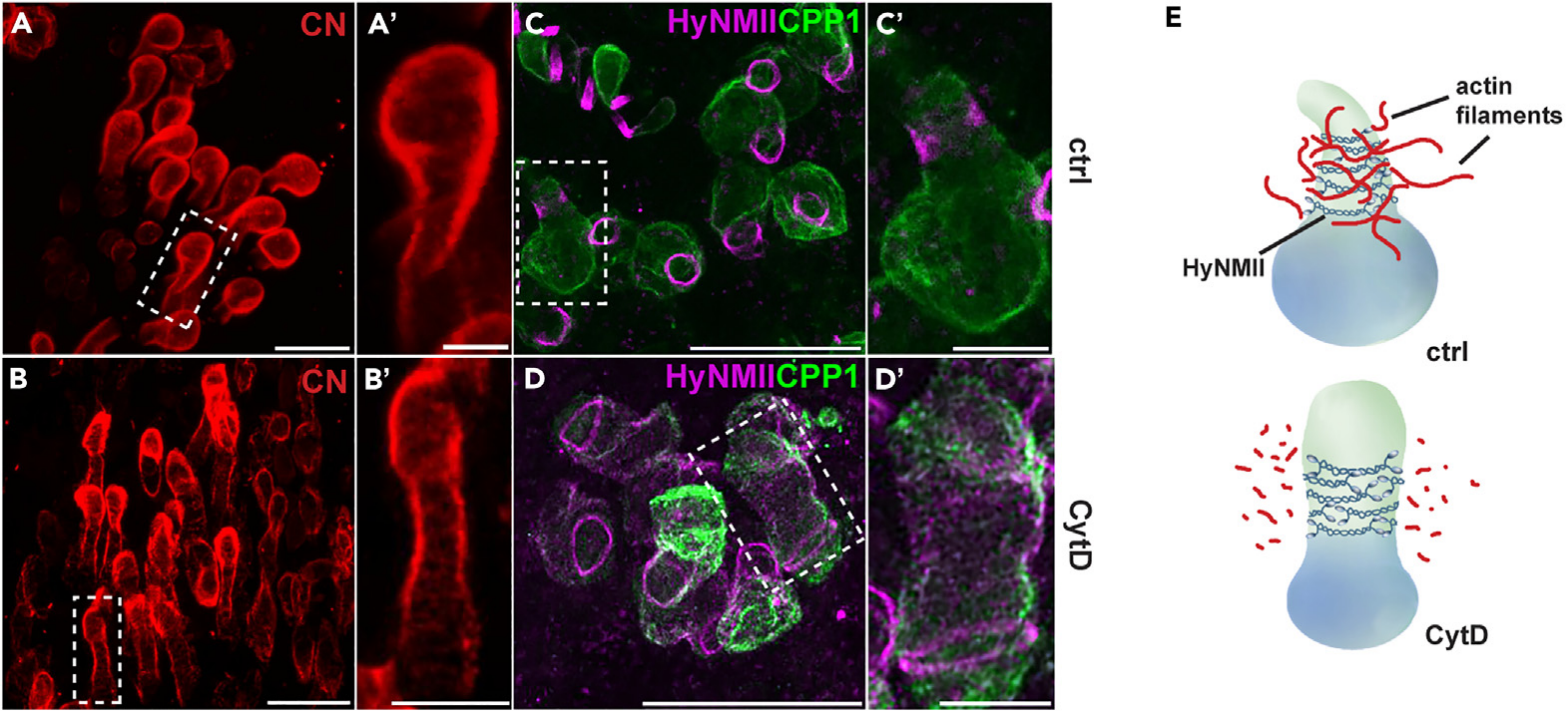
Effect of cytochalasin D treatment on nematocyst tube stability (A) Nest of stenoteles in early external tube stage stained with Cnidoin (CN) antibody showing drop-like shapes (box indicates magnified example in A′). (B) Stenotele nest at similar stage as in CytD-treated animal showing reduced capsule body sizes and expanded tube diameters (box indicates magnified example in B′). (C) Stenotele nest in early external tube stage stained with HyNMII (magenta) and CPP1 (green) antibodies showing typical narrowing at the capsule neck with a collar-like HyNMII-positive structure (box indicates magnified example in C′). (D) Stenotele nest at similar stage as in C in CytD-treated animal showing reduced capsule body and enlarged tube shaft region (box indicates magnified example in D′). Scale bars = A, B, C, D: 20 μm: A′, B′, C′, D’: 5 μm. (E) Model of the presumptive role of HyNMII and actin filaments in stabilizing by a compressive force the external nematocyst tube that collapses upon CytD treatment. See also Figure S5 for actin localization at the ultrastructural level and Figure S6 for mathematical modeling and simulations of HyNMII-induced membrane shaping.

We tested a possible role of actin in stabilizing the developing nematocyst by treating *Hydras* with cytochalasin D (CytD), an inhibitor of actin polymerization. After treating polyps for 3 h, we observed a notable effect on the shape of developing nematocysts. In early external tube stages stained with anti-Cnidoin, both, the capsule body and the tube shaft region, could be visualized (Figures 6A and 6B). While control animals showed the typical drop-like shape of the developing nematocyst with a large capsule body narrowing toward the tube’s distal end (Figures 6A and 6A′), nematocysts in cytochalasin D-treated animals had shrunken capsule bodies with tubes often exhibiting an expanding diameter toward the tip (Figures 6B and 6B′). Similar stages stained with HyNMII and CPP1 lacked the typical narrowing at the capsule neck surrounded by a HyNMII-positive collar as shown in Figures 6C and 6C’. In CytD-treated animals, the size difference between capsule body and tube was less evident, showing a markedly enlarged diameter of the HyNMII collar (Figures 6D and 6D′). Together, these observations suggest that external tube stability during development is highly sensitive to perturbations of actin. Actin depolymerization by CytD treatment apparently relaxes external tube compression leading to pressure compensation between capsule body and tube and an expansion of the tube diameter (Figure 6E).

As the earliest detection of HyNMII in developing nematocysts marks a collar-like structure at the tube base, which is still detectable in CytD-treated animals, we concluded that HyNMII concentration might correlate with membrane curvature at the tube neck region. Inspired by these findings and our hypothesis that HyNMII initially accumulates at the apical pole of the nematocyst through continuous delivery by TGN vesicles, we speculated that the membrane protrusion at the onset of tube outgrowth might be induced by HyNMII solely. This assumption is supported by a previous study demonstrating that myosin II is recruited to regions of high curvature at the myosin-binding surface of the cell membrane.^43^ Also, a recent report demonstrated that myosin-VI, favoring a saddle-shaped membrane geometry, is able to induce membrane reshaping by direct interaction with the lipid bilayer^9^; only for later processes (such as stabilizing and elongating those membrane protrusions), active forces resulting from the interplay with actin would come into play.^9^ We investigated this possibility by applying a mathematical model first proposed for ESCRT-molecules, in which a mechanism called “bud-neck scaffolding” has been shown to initiate membrane budding by locally creating saddle-shaped membrane curvatures (i.e., regions with negative Gaussian curvature)^44^ (Figures S6A–S6F). In the simulation approach, the membrane is described as a continuous 2D surface curved in 3D space, and lateral protein concentrations are given by an order-parameter. Here, proteins can on the one hand induce local mean- and Gaussian curvatures (Figures S6D–S6F), and on the other hand, they show the tendency for clustering.

Our simulations demonstrate that a saddle-shaped HyNMII is sufficient to spontaneously induce membrane structures strongly resembling those observed during early external tube formation (Figures 1H, 1K, 1N, and S6A–S6C). In particular, a “pointed hat”-like geometry of the membrane is formed, where HyNMII forms a broad belt around the base of the protrusion but is missing at the tip and in the surrounding membrane. Importantly, this process is simulated as a pure passive (energy minimizing) process, solely driven by the interplay of protein shape and -sorting with the membrane curvature, without any contribution of active (e.g., “pushing”) forces as induced by actin. We propose that the latter process exerts a compressive force on the tube countering the osmotic pressure of the capsule and thus stabilizing its elongation phase (Figure 6E).

## Discussion

The cnidarian stinging organelles, nematocysts or cnidocysts, are the hallmark of the cnidarian clade and represent one of the most sophisticated subcellular structures in the animal kingdom. A recent study has detailed the mechanism of nematocyst discharge in the anthozoan *N. vectensis* at high resolution, emphasizing the role of the complex tube shaft region for the initial steps of tube expulsion.^45^ The formation of the tube or thread and its invagination process constitute the most elaborate part of nematocyst development, and tube morphology, including the adornment with spines, does not only vary between cnidarians but also between nematocyst types of the same species. While early ultrastructural studies have emphasized the role of the microtubule cytoskeleton for the structural integrity of the nematocyst^46^ and the formation of the external tube,^17^^,^^32^ actomyosin networks have so far not been implicated in nematocyst morphogenesis. Here, we demonstrate the assembly of HyNMII at the cytoplasmic face of the nematocyst driving the early apical protrusion and subsequent elongation of the external tube as key steps in nematocyst development. Our data indicate that HyNMII already plays a role in the formation of transport vesicles targeting the growing nematocyst, which is supported by several reports addressing the role of myosin II in vesicle fission from the Golgi and intracellular membrane trafficking.^1^^,^^7^^,^^47^^,^^48^ We assume that HyNMII is enriched at the apical pole of the nematocyst by fusion of HyNMII-coated TGN vesicles with the nematocyst membrane. Vesicle transport to the nematocyst is organized by a crest of microtubules covering the tube tip, and it has been shown that the number of microtubules is adjusted to the tube diameter, which varies in different nematocyst types.^17^ We therefore propose that the cytoskeletal organization of the nematocyst-associated TGN creates a kind of “imprint” of HyNMII protein at the nematocysts’ apical pole, which then defines the tube diameter by imposing a membrane curvature at this position. Our mathematical model suggests that this initial process is driven by a critical concentration of HyNMII, which assembles to a collar structure reflecting the circular arrangement of the TGN microtubules. The interaction of myosin II with Golgi membranes is usually described as a dynamic process, regulated by G-proteins and to be highly sensitive to brefeldin A.^1^ The fact that HyNMII remains attached to the tube during invagination, which involves a release of the tube from the nematocyst membrane, is remarkable, as it implies a re-localization of HyNMII from the cytoplasmic face of the membrane to the capsule lumen. As this process is connected to the retraction of the membrane originally enclosing the tube, it might be associated with exocytosis into the nematocyst matrix. A similar mechanism has been described for membrane dynamics during cell migration in which the retracting membrane is removed by endocytosis.^49^

We assume that the contractile activity of HyNMII in concert with the actin cytoskeleton stabilizes and shapes tube elongation by a compressive force (Figure 6E). Although the increase of osmotic pressure in the nematocyst associated with poly-γ-glutamate synthesis^24^ has been described to occur mostly after tube invagination, we cannot exclude that low poly-γ-glutamate concentrations in the early differentiation phase might generate a pushing force on the tube membrane which could be controlled by the outer HyNMII sheath. This would explain the enlarged tube diameters by CytD (Figures 6A–6E) and the bulges appearing mostly at tube tips already in the early phase of BBS treatment (Figures 2B′–2D′). Prolonged BBS incubation then leads to a detachment of HyNMII-rich particles from the developing nematocyst, plausibly interpreted as a result of tube disintegration (Figures 5J and 5K). Similar effects of BBS treatment on organelle or cell shape integrity have been described in other systems, such as in nuclear flattening during cell proliferation^50^ and in red blood cell membrane curvature.^51^

Tube invagination and maturation, including the assembly of spines, constitute the most remarkable phase of nematocyst development. The spines form inside the tube after invagination and are then turned to the outside during discharge. Spine proteins like spinalin,^52^^,^^53^ which accumulate to large granules in the capsule matrix during early morphogenesis^19^ therefore have to pass the tube wall to reach the prospective outer surface of the tube. We assume that NOWA takes a similar route from the capsule matrix, probably at the fold where the tube is bent inwards (Figure 4M). This would ensure a continuous feeding of NOWA protein into the lumen of the incoming tube to drive its invagination. Interestingly, the NOWA spots observed in the capsule matrix locally showed a transient, lattice-like structure in transmission EM (Figures 4H and 4I). We have demonstrated previously that recombinant NOWA forms disulfide-linked oligomers under oxidizing conditions via its repetitive cysteine-rich domains (CRDs) at the protein’s C-terminus.^54^ A shift in the redox potential of the capsule matrix might therefore be responsible for the observed dissociation of the NOWA spots concomitant with tube invagination (Video S1, Figure S3). The unusually high number of CRDs (8) probably ensures that NOWA stays oligomeric to some extent. It is an attractive hypothesis that the multiple lectin domains presented by such oligomers cross-link the inner surfaces of the incoming tube by an aggregation-type cluster glycoside effect.^55^ This multivalent protein-carbohydrate interaction could induce a zipper-like mechanism facilitating invagination by an ATP-free process (Figure 4M).

Our study addresses critical steps in the morphogenesis of the nematocyst. It elucidates a hitherto unknown role of the actomyosin cytoskeleton in tube formation as a conserved process in cnidarians and, by revising the role of NOWA using transgenic and genetic knockdown approaches that were previously not available, offers a molecular model for the mechanism of tube invagination. Taken together, our data underscore the unique complexity in the molecular assembly and morphogenesis of the cnidarian stinging organelle.

### Limitations of the study

Our study focuses on the model organism *Hydra*, and we have so far limited evidence whether tube formation in other cnidarian species follows the same molecular principles. In addition, in several experiments, only stenoteles were chosen as representative nematocyst type due to their large size, which facilitates imaging.

The presented model demonstrates phenomenologically that a certain effective protein shape is capable of inducing mechanochemical membrane patterns experimentally observed during early stages of nematocyst development. For a deeper understanding of the process, it should be investigated how myosin II (possibly in interplay with additional proteins) could induce the assumed effective shape. In addition, only passive (i.e., energy minimizing) processes are modeled. In order to understand later stages of nematocyst development, also active forces (e.g., inducing tube growth) should be considered as well.

The microscopic documentation of actin’s spatial organization in nematocytes is certainly incomplete. Fluorescence light microscopy of whole-mount *Hydra* specimens does not sufficiently resolve delicate meshworks of single actin filaments, as apparently prevailing in developing nematocytes. Electron microscopy, optionally combined with immunogold techniques, frequently fails to visualize or to label those fine structures embedded within the nematocytes’ cytoplasm that is particularly densely packed with abundant ER and ribosomes, as detailed in the results section. To cope with those cell-specific properties and to overcome the associated technical problems (i.e., steric hindrance preventing antibody binding in well-preserved samples), further methodological improvements are needed for eventually obtaining comprehensive insight into the spatial organization of actin and its relation to HyNMII during nematocyte development.

## STAR★Methods

### Key resources table

**Table:** 

REAGENT or RESOURCE	SOURCE	IDENTIFIER
**Antibodies**		

Alexa Fluor® 488 goat anti-rat IgG (H+L)	Thermo Fisher Scientific	Cat# A-11006; RRID: AB_2534074
Alexa Fluor® 568 goat anti-rabbit IgG (H+L)	Thermo Fisher Scientific	Cat# A-11011; RRID: AB_143157
Alkaline phosphatase (AP)-conjugated anti-DIG antibody	Roche	Cat# 11093274910
Goat Anti-Guinea Pig IgG (H+L) Highly Cross-adsorbed Antibody, Alexa Fluor 488 Conjugated	Molecular Probes	Cat# A-11073; RRID: AB_2534117
Goat anti-rabbit/-mouse IgG (H+L) colloidal gold	British Biocell	Cat# EM.GAFAR10/EM.GFAF5
Guinea pig anti-Cnidoin	Eurogentec	N/A
Mouse anti-actin	Chemicon	Clone C4; MAB1501R; RRID:AB_222304
NANOGOLD-Fab′goat anti-rabbit/-mouse IgG (H + L)	Nanoprobes Yaphank	Cat# 2004; RRID:AB_2631182; Cat#2002
Peroxidase AffiniPure Goat Anti-Rabbit IgG (H+L)	Jackson ImmunoResearch Labs	Cat# 111-035-003; RRID: AB_2313567
Rabbit anti NOWA-CTLD	Eurogentec	N/A
Rabbit anti-HyNMII	Eurogentec	N/A
Rabbit anti-Ncol-1pp	Eurogentec	N/A
Rat anti-CPP-1	Eurogentec	N/A

**Bacterial and virus strains**		

*E. coli* BL21 (DE3)	Thermo Fisher Scientific	Cat# C600003

**Chemicals, peptides, and recombinant proteins**		

Acetic acid	Fluka™, Honeywell	N/A
Albumin Fraktion V, NZ-Origin (BSA)	Carl Roth	N/A
Antigenic peptide HyNMII: LSQLYKEQLQGLMNTL	Eurogentec	N/A
4′,6-Diamidine-2′-phenylindole dihydrochloride (DAPI)	Roche Diagnostics	N/A
(-)-Blebbistatin	Sigma-Aldrich	Cat# 203391; CAS 856925-71-8
Bovine serum albumin	Sigma-Aldrich	Cat# A-9647
CaCl_2_	Carl Roth	Cat# 5239.1; CAS 10035-04-8
CHAPS	Sigma-Aldrich	Cat# C3023
Cytochalasin D	EMD Millipore Corp (Affiliate of Merck KGaA)	Cat# 250255; CAS 22144 77-0
Denhardt′s Solution	Sigma-Aldrich	Cat# D2532
Dimethylsulfoxid	Thermo Fisher Scientific	Cat# D12345
Dithiothreitol	Sigma-Aldrich	Cat# D9779
Formaldehyde solution min. 37%	Merck KGaA	N/A
D-(+)-Glucose	Sigma-Aldrich	Cat# G8270
Glutaraldehyde	Sigma-Aldrich	Cat# G5882
Glycerol 99.5% bidistilled	VWR International	N/A
Glycine	neoFroxx	N/A
Heparin sodium salt from porcine intestinal mucosa	Sigma-Aldrich	Cat# H3149
HQ-Silver™	Nanoprobes Yaphank	Cat# 2012
Isopropyl-β-D-1-thiogalactopyranosid	Sigma-Aldrich	Cat# I6758
Kanamycin	Sigma-Aldrich	Cat# K0254
KCl	Sigma-Aldrich	Cat# P9541
Methanol ≥99.8%	Riedel-de Haën™, Honeywell	N/A
Methyl cellulose	Sigma-Aldrich	Cat# M0512
MgSO4	Sigma-Aldrich	Cat# M7506
Mowiol	Sigma-Aldrich	N/A
NaCl	neoFroxx GmbH	N/A
Na_2_HPO_4_ ∗ 2 H_2_O	Fluka™	N/A
KCl	AppliChem GmbH	N/A
KH_2_PO_4_	Chemsolute, Thomas Geyer	N/A
Osmium tetroxide	Sigma Aldrich	Cat# O5500
Oxalic acid	Sigma-Aldrich	Cat# O-0376
Paraformaldehyde	Carl Roth	N/A
Percoll™	GE Healthcare Bio-Sciences AB	N/A
Phosphotungstic acid hydrate	Sigma-Aldrich	Cat# P79690
2-Propanol ≥99.8%	Riedel-de Haën™, Honeywell; https://lab.honeywell.com/shop/2-propanol-33539	N/A
2-Propanol 99.9% (for cleaning)	Zentralbereich Heidelberg University; http://znfshop.zbt.uni-heidelberg.de/2-propanol-99-9-isopropanol-rein.html	N/A
(-)-Linalool	Sigma-Aldrich	Cat #W263516; CAS 126-91-0
Rifampicin	Sigma-Aldrich	Cat# R3501
D(+)-Saccharose	Carl Roth	N/A
Sodium citrate	Sigma-Aldrich	Cat# 1613859
Sodium pyruvate	Sigma-Aldrich	Cat# P2256
Streptomycin	Sigma-Aldrich	Cat# 85886
Triethanolamine	Sigma-Aldrich	Cat# T58300
Triton^TM^ X-100	Sigma-Aldrich	Cat# 9036-19-5; X100PC-5ML
TES	Carl Roth	Cat# 9137.1
Tween®-20	Sigma-Aldrich	Cat# 8.17072
Uranyl acetate	Electron Microscopy Sciences	CAS #541-09-3

**Critical commercial assays**		

Plasmid Mini Kit	QIAGEN	Cat# 12123
Plasmid Midi Kit	QIAGEN	Cat# 12143
DIG RNA labelling Kit (SP6/T7)	Sigma-Aldrich	Cat# 11175025910

**Experimental models: Organisms/strains**		

*Hydra magnipapillata strain 105*	Toshitaka Fujisawa lab, National Institute of Genetics, Mishima, Japan	N/A
*Hydra* vulgaris AEP	Thomas Bosch lab, University of Kiel, Germany	N/A
*Artemia salina* nauplii	Tropic Marine	N/A
*Hydra* vulgaris AEP NOWAp::NOWA:GFP	This study	N/A
*Hydra vulgaris* AEP Act::GFP^ectoderm^/Act::RFP^endoderm^	Dr. Robert Steele	N/A
*Hydra vulgaris* AEP Cnnos1::GFP	Dr. Chiemi Nishimiya- Fujisawa, National Institute of Genetics, Mishima, Japan	N/A

**Oligonucleotides**		

siRNA targeting sequences: HyNMII: 1. AAGUUCAAUUGCAGCUUGAUA 2. AAAGACAAAAGUUGCAACUCG	Sigma-Aldrich	N/A
siRNA targeting sequences: NOWA: 1. AACGAAGUCAUGUGCAUCAUA 2. AACGAAGUCAUGUGCAUCAUA	Sigma-Aldrich	N/A
siRNA targeting sequence: GFP: AAGGUGAUGCAACAUACGGAA	Sigma-Aldrich	N/A

**Recombinant DNA**		

Plasmid: NOWA::NOWA-GFP	This paper	N/A
Plasmid: Actin::GFP	This paper	N/A
Plasmid: pet19b-HyNMII motor domain	This paper	N/A

**Software and algorithms**		

Image J version 2.1.0/1.53c	Open-source image processing software	https://imagej.nih.gov/ij/ RRID:SCR_003070
Bio-formats plugin (FIJI)	Opensource	RRID:SCR_000450
NIS elements Imagine software	Nikon Instruments Inc.	https://www.microscope.healthcare.nikon.com/products/software/nis-elements
Microsoft Word version 16.64	Microsoft	https://www.microsoft.com/
Microsoft excel version 16.63.1	Microsoft	RRID:SCR_016137; https://www.microsoft.com/
Inkscape version 1.0.2	Opensource	https://www.inkscape.org
Geneious Prime version 2020.2	Dotmatics	https://www.geneious.com/
Python version 3.9.7	Python Software Foundation	https://www.python.org/
Pandas version 1.4.3	Opensource	https://pandas.pydata.org/
iTEM	EMSIS	https://www.emsis.eu
Photoshop CS6 23.5.0	Adobe	https://www.adobe.com
Adobe Illustrator 26.5	Adobe	https://www.adobe.com
Prism 9	Graphpad	https://www.graphstats.net/

**Other**		

Cover slips 24x32 mm	Paul Marienfeld	N/A
Disposal bags	Sarstedt	N/A
Double-sided adhesive tape	Tesa SE	N/A
KL1500 HAL Light source for stereo microscopy	Schott AG	N/A
Microscope slide 76x26 mm	neoLab Migge	N/A
Pasteur capillary pipettes	neoLab Migge	N/A
Roller mixer	Ingenieurbüro CAT, M. Zipperer	N/A
Stereo microscope	Nikon Minato, Tokyo, Japan	N/A
Platform shaker	Heidolph	N/A
Test tube shaker	REAX 1 R, Heidolph	N/A
Whatman™ 3 MM CHR	GE Healthcare UK Limited	N/A
Whatman® lens cleaning tissue, Grade 105	Whatman, Cytiva	N/A
Precast 4-15 % gradient gels	Carl Roth	Cat #3673.2
Nikon A1R Confocal Laser Scanning Microscope	Nikon, Tokyo, Japan	RRID:SCR_020317
Nikon Ti2 microscope	Nikon, Tokyo, Japan	RRID:SCR_021068
Zyla 4.2 CMOS camera	Oxford Instruments Andor	N/A
Nikon Eclipse 80i microscope	Nikon Eclipse 80i Advanced Research Microscope	RRID:SCR_015572
Gene Pulser® Cuvette, 0.4 cm electrode gap	Bio-Rad	Cat# 1652088
GenePulser XcellTM	Bio-Rad	Cat# 1652660

### Resource availability

#### Lead contact

Further information and request for resources and reagents should be directed to and will be fulfilled by the lead contact, Suat Özbek (suat.oezbek@cos.uni-heidelberg.de).

#### Materials availability

All unique/stable reagents and animal strains generated in this study are available upon request from the lead contact, Suat Özbek (suat.oezbek@cos.uni-heidelberg.de).

### Experimental model and subject details

#### Animals

*Hydra magnipapillata* (105) is a clonal line originally described by Sugiyama & Fujisawa^56^ and obtained from the Toshitaka Fujisawa lab, National Institute of Genetics, Mishima, Japan. *Hydra vulgaris* (AEP) was first described in^57^ and obtained from the Thomas Bosch lab, University of Kiel, Germany, where it was used to create the first transgenic Hydra strain.^58^ All wildtype and transgenic *Hydra* strains were kept in at 18°C in *Hydra* medium (HM, 1 mM CaCl_2_, 1 mM NaH_2_CO_3_, 0.1 mM MgCl_2_, 0.1 mM KCl, pH 6.8) and fed two to three times per week with freshly hatched *Artemia salina* nauplii. The medium was renewed 3–4 h after feeding and again the following day. Prior to each experiment animals were starved for at least 24 hours.

#### Transgenesis

The transgenic NOWA::NOWA-GFP vector was designed on the basis of the hOT G construct expressing eGFP under control of the *Hydra* actin promotor provided by the Thomas Bosch lab at Kiel University.^58^ For this, the actin promotor sequence was replaced by 1127bp of the NOWA 5′ region preceding the open reading frame using KpnI and BamHI restriction sites, respectively. In the expression construct the eGFP sequence is flanked by the NOWA signal peptide at the 5′ end and the full-length NOWA sequence lacking the signal peptide at the 3′ end. The coding sequence of the eGFP-NOWA fusion is followed by the actin 3′ UTR. Generation of the transgenic NOWA::NOWA-GFP line was performed by microinjection of the expression vector into embryos removed from *H. vulgaris* (AEP) females at the two- to eight-cell stage.^58^ Fully transgenic animals were obtained by random budding.

### Method details

#### Inhibitor treatments

(-)-Blebbistatin was solubilized in DMSO (dimethylsulfoxid) at 17 mM and stored at -20°C. For the treatment, the stock solution was diluted in HM to 0.25 μM. For controls, DMSO was dissolved in HM to the corresponding concentrations. Blebbistatin treatment was performed continuously for 7 days and animals were kept at 18°C in 25 mL Petri dishes with daily exchange of medium. At least 10 animals were treated and analyzed per treatment time and concentration. Cytochalasin D was solubilized in DMSO to prepare a stock solution of 1 mg/mL and stored at -80°C. For treatment, the stock solution was diluted to 5 μg/mL and 10 animals were incubated with the inhibitor in 2 mL Eppendorf tubes at RT on a shaker for 3 hours and fixed for antibody staining.

#### Immunohistochemistry

For whole mount immunostainings, animals were starved for 24 hours and relaxed in 2 % urethane for 1-2 minutes. Mainly two fixatives were used – buffered, freshly prepared 4 % paraformaldehyde (Ncol-1pp, NOWA-CTLD, Cnidoin) or Lavdovsky’s fixative (50 % ethanol, 10 % formaldehyde, 4 % acetic acid) (HyNMII, CPP1) - for 30 minutes at room temperature or overnight. After incubation with the fixative, animals were washed in subsequent steps starting with PBS/0.1 % Tween-20, followed by PBS/0.1 % Triton X-100 and again with PBS/0.1 % Tween-20. All washing steps were done three times with an incubation time of 10 minutes between each step. Animals were then incubated over night at 4°C with the primary antibodies in PBS/0.1 % Tween-20/ 0.5 % bovine serum albumin (BSA). Following dilutions were used: NOWA-CTLD (rabbit) 1:400, HyNMII (rabbit) 1:100; CPP1 (rat) 1:100; Ncol1pp (rabbit) 1:100. In the next step, the primary antibodies were washed using PBS/0.1 % Tween-20, three times with 10 minutes incubation. Secondary antibodies were diluted 1:400 in PBS/0.1 % Tween-20/1 % BSA. Animals were incubated for 2-3 hours with this diluted secondary antibody, followed by addition of DAPI (1:1000) for nuclei staining and incubated for 10 minutes. Animals were washed in PBS and mounted on clean glass slides with spacers using Mowiol and sealed with a cover slip. For peptide competition, the antigenic HyNMII peptide used for immunization was added at 1 mg/mL during primary antibody incubation. For the transgenic NOWA::NOWA-GFP strain, the living animals were imaged without any fixation and mounted either in 2 % agarose or 90 % glycerol. For this, approximately 5 adult animals were collected and relaxed by incubating in 2 % Urethane in HM for 5 minutes or in Linalool (1 μM prepared in HM) for 15 minutes. Next, the samples were washed once with PBS, mounted on slides with spacers in 90 % Glycerol, covered with a cover slip and sealed with nail polish. The prepared slides were imaged directly after mounting. For mounting in 2 % agarose, the collected animals were relaxed with Linalool for approximately 10 minutes. The foot and tentacles were cut off and the remaining body columns were transferred on the slides with double-sided tape spacers. After removing the remaining medium, 2 % low gelling temperature agarose with 1:1000 Linalool was added and the slides were sealed with a coverslip. The samples were imaged on the same day. The images were acquired with the following microscopes: Nikon A1R confocal laser scanning microscope using the NIS Elements software, Versions 4.51.01 or 5.11.01 and objectives Apo LWD 40x WI λS (NA 1.1), Apo 60x Oil λS (NA 1.4), and Plan Apo λ 20x (NA 0.75). Alternatively, a Nikon AX microscope using the NIS Elements software, Version 5.40.02 and the objectives mentioned above. Further image processing was performed with Adobe Photoshop CS6 and Fiji.

#### *Hydra* live imaging

NOWA::NOWA-GFP animals were starved for one week before live imaging. During mounting and imaging, they were relaxed in 1μM Linalool^59^ in *Hydra* medium and tentacles and foot were cut off to minimize animal movement. The body columns were mounted in coverslip bottom dishes and fixed with 2% ultra-low gelling temperature agarose. A coverslip was mounted with spacers on top of the animals/agarose to limit evaporation of linalool during imaging. Time lapse imaging was performed on a Nikon Ti2 microscope equipped with a LWD 40x NA 1.1 water immersion objective and water dispenser. eGFP signal was captured on a Andor Zyla CMOS camera in wide field mode. A z-stack of 20 μm with 2 μm spacing was imaged every 8 minutes over 10 hours at low illumination and resulting images were denoised by Nis-Elements 5.2. The movie shows a projection of 3 slices.

#### Electron microscopy (EM)

EM in Figure 3B was performed as described in.^17^ EM in all other figures was performed according to established standards,^60^^,^^61^ but with slight, specifically tailored adaptations. For morphology, intact polyps were rapidly cryoimmobilized through means of high-pressure freezing, followed by freeze-substitution at −90°C overnight (with acetone containing 1% (w/v) OsO_4_, 0.1% (w/v) uranyl acetate and 4% (v/v) water) and sample warming to room temperature. After several rinses with acetone samples were embedded in epoxy resin. Ultrathin sections were optionally poststained with heavy metals (Reynolds’ lead citrate^62^; or 1% (w/v) phosphotungstic acid in 95% (v/v) ethanol, 5 min at +60°C to highlight the cytoskeleton and membranes^63^). Immuno-EM comprised both pre- and post-embedding labelling techniques,^60^ depending on the antigen. For pre-embedding NANOGOLD™ immunolabelling of HyNMII and actin in developing nematocytes strong permeabilization, performed simultaneously with chemical fixation, proved indispensable (note that those protocols are not standard for immuno-EM because of the limited ultrastructure preservation). HyNMII detection required specimen fixation with 80% (v/v) methanol plus 20% (v/v) DMSO^64^ applied for 30 minutes at −20°C, whereas actin in the cytoplasm of nematocytes was detected after specimen fixation with 4% (w/v) formaldehyde in 0.1M phosphate buffer, mixed with 0.5% (v/v) Triton^TM^ X-100 (peroxide- and carbonyl-free) for 30 minutes at room temperature (modified after^65^). Fixed samples were stored overnight at +4°C in 4% buffered formaldehyde. Subsequent pre-embedding NANOGOLD™ immunolabelling was performed at room temperature according to established standards.^60^ Samples were rinsed for 30 min with 0.5% (v/v) Triton^TM^ X-100 in PBS, incubated for 30 min with 0,05M glycine plus 0.5 % (v/v) Triton^TM^ X-100 in PBS to block free aldehydes, followed by incubation for 30 min with 5 % (w/v) bovine serum albumin plus 0,1 % (v/v) cold water fish gelatin and 0.5 % (v/v) Triton^TM^ X-100 in PBS to prevent unspecific antibody binding. Primary and secondary antibodies were diluted in incubation buffer made of 0.1 % (v/v) bovine serum albumin plus 0.5 % (v/v) Triton^TM^ X-100 in PBS and applied overnight. Bound rabbit anti-HyNMII (1:100) or mouse anti-actin (1:250) were visualized with NANOGOLD-Fab′goat anti-rabbit/-mouse IgG (H + L) (diluted at 1:150), followed by 15 min postfixation with 2.5 % (v/v) glutaraldehyde, rinsing for 45 min with double distilled water, silver enhancement of the 1.4 nm small NANOGOLD™ conjugates with HQ-Silver (8 min at room temperature), and epoxy resin embedding. Post-embedding indirect immunogold labelling with rabbit anti-NOWA-CTLD (diluted 1:100) or with mouse anti-actin was performed on thawed ultrathin cryosections from samples fixed for 30 minutes at room temperature with 8 % (w/v) formaldehyde in 0.1 M phosphate buffer and cryoprotected overnight with 2.3 M sucrose in PBS according to established standards.^60^ The general incubation procedure was the same as for the preembedding approach, except that no Triton^TM^ X-100 was added and bound primary antibodies were visualized by secondary antibodies coupled to 5 or 10 nm colloidal gold (without any silver enhancement). Postfixed and rinsed samples were stained for 5 min with 2 % (w/v) neutral uranyl acetate in 0.15 M oxalic acid and embedded in 2 % (w/v) aqueous methylcellulose containing 0.4 % (w/v) uranyl acetate. Samples were analyzed with a CM120 TEM (Philips, Eindhoven, The Netherlands) equipped with a MORADA G1 digital camera (EMSIS, Münster, Germany). Size measurements were done with iTEM software, Photoshop CS6 was used for adjusting brightness, greyscale, and sharpness of the digital images, as well as for pseudo coloring.

#### siRNA knockdown by electroporation

siRNA knockdown was performed by electroporation using a transgenic *Hydra* vulgaris strain expressing endodermal RFP and ectodermal GFP.^66^ Animals from a daily-fed culture were washed twice with ultrapure water. For each reaction, 20 animals were transferred to electroporation cuvettes (4 mm gap, Bio-Rad) and excess liquid was removed. After adding 200 μl of sterile ultrapure water containing either 3 μM of siGFP or a combination of siGFP and two different target siRNAs (1 μM each) animals were allowed to relax for 20 min at room temperature. Thereafter, a single square pulse at 250 V was administered for 25 ms using a GenePulser XcellTM (Bio-Rad) electroporation system equipped with a CE module. Immediately after the pulse, 500 μl restoration medium consisting of 80% HM and 20% hyperosmotic dissociation medium (6 mM CaCl2, 1.2 mM MgSO4, 3.6 mM KCl, 12.5 mM TES, 6 mM sodium pyruvate, 6 mM sodium citrate, 6 mM glucose and 50 mg/l rifampicin, 100 mg/l streptomycin, 50 mg/l kanamycin, pH 6.9) was added to the cuvette, the animals were transferred to Petri dishes containing restoration medium and allowed to recover for one day. Viable polyps were transferred to new dishes containing HM and maintained under standard culture conditions.

#### Recombinant protein expression and Western blot

The cDNA coding for the HyNMII motor domain (residues 84-760) was amplified by PCR and cloned via a NdeI site into the pet19b vector (Novagen), which introduces an N-terminal polyhystidine tag. The recombinant protein was expressed in *E. coli* BL21 (DE3) cells by Isopropyl-β-D-1-thiogalactopyranosid induction and the bacteria were lysed by ultrasound treatment and several freeze/thaw cycles. The obtained pellets containing the motor domain protein in inclusion bodies were washed 3 times using PBS/Tween-20 and then solubilized in protein sample buffer for SDS-PAGE using precast 4-15 % gradient gels. The proteins were transferred to PVDF membranes by wet blotting. Membranes were blocked for 1 hr at RT in PBS containing 5 % BSA and 0.2 % Tween-20 (PBST), incubated with polyclonal rabbit HyNMII antibody at 1:500 in 1 % BSA at 4°C overnight, washed 3 × 5 min with PBST, and then incubated with anti-rabbit horseradish peroxidase-conjugated antibody at 1:10,000 in PBST containing 5 % BSA for 1 hr at RT. The membrane was washed 2 × 5 min with PBST and 2 × 5 min with PBS and blots were developed using a peroxidase substrate for enhanced chemiluminescence. For peptide competition, the antigenic peptide used for immunization was added at 1 mg/mL during primary antibody incubation. For Western blot detection of NOWA-CTLD nematocysts were isolated from wild-type and NOWA::NOWA-GFP transgenic animals by gradient centrifugation.^67^ For this, about 200 polyps were washed in ice-cold water and frozen at -20°C. After thawing the polyps were gently homogenized with a pasteur pipette in an ice-cold sucrose (300mM) solution containing 50 % Percoll (v/v). The homogenates were then centrifuged for 10 min at 3000 g and the supernatant which contained debris and cell fragments was discarded. The pellet consisting of intact non-discharged nematocysts was resuspended and washed several times in the sucrose solution. 2 x 10^5^ isolated nematocysts of each preparation were incubated for 30 minutes at 96°C with 1M dithiothreitol in PBS to dissociate the capsule polymer. After addition of 3 μl protein sample buffer for SDS-PAGE the nematocysts were incubated for 20 minutes at 96°C and afterwards centrifuged at 13,300 rpm for 5 minutes. SDS-PAGE and Western blotting were performed as described above. The membrane was incubated overnight with the primary NOWA-CTLD antibody at 1:2000 and secondary anti-rabbit-HRP antibody at 1:10000.

#### *In situ* hybridization

For WISH, animals were relaxed in 2% urethane for 1–2 minutes in HM and fixed overnight at 4°C in 4 % formaldehyde and cleared in two changes of 100 % methanol for 10 min each and stored at −20°C until further use. To block endogenous peroxidase cleared animals were incubated for 30 min in methanol containing 1 % hydrogen peroxide. Animals were then rehydrated through a graded series of methanol (100%, 75%, 50%, and 25%) in PBT (phosphate-buffered saline with 0.1% Tween-20) for 5 minutes each and rinsed three times for 5 minutes each in PBT. After digestion for 7 minutes with 10 mg/ml proteinase K in PBT, animals were washed in 4 mg/ml glycine in PBT for ten minutes, glycine was removed by two 5-minute washes in PBT, and samples were treated twice for 5 minutes with 0.1 M TEA (triethanolamine) and 5 minutes with 0.25 % (v/v) acetic anhydride in 0.1 M TEA, followed by 0.5 % (v/v) acetic anhydride in 0.1 M TEA. After two further washes in PBT, samples were fixed for 20 minutes in 4 % formaldehyde in HM at RT, then washed five times for 5 minutes in PBT. Samples were washed for 10 minutes in 50 % PBT/50 % hybridization solution (1:1 mixture of deionized formamide and buffer containing 5x saline-sodium citrate (750 mM NaCl, 75 mM sodium citrate), 0.2 mg/ml yeast tRNA, 2 % of 50x Denhardt’s solution, 0.1 mg/ml heparin, 0.1 % Tween-20 and 0.1 % CHAPS). DIG (Digoxygenin)-labeled RNA probes were prepared using the DIG-RNA Labeling Kit. The RNA fragment used corresponds to the tail of HyNMII (bp 3720-4747). For hybridization each probe was added at a final concentration of 0,025 ng/mL for 60 hours at 55°C. After washing, samples were incubated overnight in with AP (alkaline phosphatase)-conjugated anti-DIG antibody. Undiluted BM purple was used as color reaction substrate. The reaction was stopped after incubation at RT for 13 minutes. Whole animals were rehydrated and mounted on object slides in PBS and 90 % glycerol. Images were captured using a Nikon Eclipse 80i microscope.

#### Mathematical modelling

To investigate if (passive) bud-neck scaffolding due to myosin II is sufficient to explain early steps of nematocyst development, we adopted the membrane simulation framework of.^44^ In particular, membrane protein shapes can be described by two geometric measures: the local mean curvature as well as the Gaussian curvature, which can describe a multitude of effective protein shapes (Figure S6D). Mechanical membrane energy is governed by a modified Helfrich energy, and membrane deformations as well as lateral dynamics are driven by passive gradient flows of the combined Helfrich- and Cahn-Hilliard type energy. More technical details of model derivation and numerical implementation are given in.^44^ Simulations have been performed on a square of 3 x 3 numerical units with periodic boundary conditions, using an initial Gaussian distribution of both, initial membrane height values as well as the initial HyNMII concentration (Figure S6A). As parameters we prescribed a preferred mean curvature of H_0_=2 and K_0_=-4.5 for myosin II, meaning that this molecule prefers saddle-shaped regions^9^ (negative K_0_-value) on the one hand, but positive mean curvatures on the other hand. This choice was mainly motivated by the expected curvature in experimentally observed early “pointed-hat” stages (Figures 1H, 1K, 1N, 6C, and 6C′). For the Cahn-Hilliard parameter Xi we chose 0.15, all other parameters have been set to the values used in.^44^

### Quantification and statistical analysis

The statistical analysis corresponding to Figures 2F, S2D, and 3M was based on one-way ANOVA test and an unpaired t-test was employed for Figure 4I. We used Prism to perform statistical analysis. Raw data can be found in Tables S1–S5. Data quantification was done manually and accounted for in Microsoft Excel spreadsheets. Further details of the quantification and statistical methods are described in the corresponding figure legends. The rain cloud plot in Figure 2G was made with the help of python libraries ‘pandas’, ‘ptitprince’, and ‘matplotlib’.

## Data Availability

Data have been deposited in the supplemental information and details are listed in the key resources table. This paper does not report original code. Any additional information required to reanalyze the data reported in this paper is available from the lead contact upon request.
