# Supplementary material for: Non-muscle myosin II drives critical steps of nematocyst morphogenesis
Source: iScience. 2023 Feb 28;26(3):106291. doi: 10.1016/j.isci.2023.106291 (PMC10014300; doi:10.1016/j.isci.2023.106291)
Supplement: Document S1. Figures S1–S6 and Tables S1–S5 [file mmc1.pdf]

iScience, Volume *26*

Supplemental information

Non-muscle myosin II drives

critical steps of nematocyst morphogenesis

Niharika Garg, Urška Knez Štibler, Björn Eismann, Moritz Mercker, Bruno Gideon Bergheim, Anna Linn, Patrizia Tuchscherer, Ulrike Engel, Stefan Redl, Anna Marciniak-Czochra, Thomas W. Holstein, Michael W. Hess, and Suat Özbek

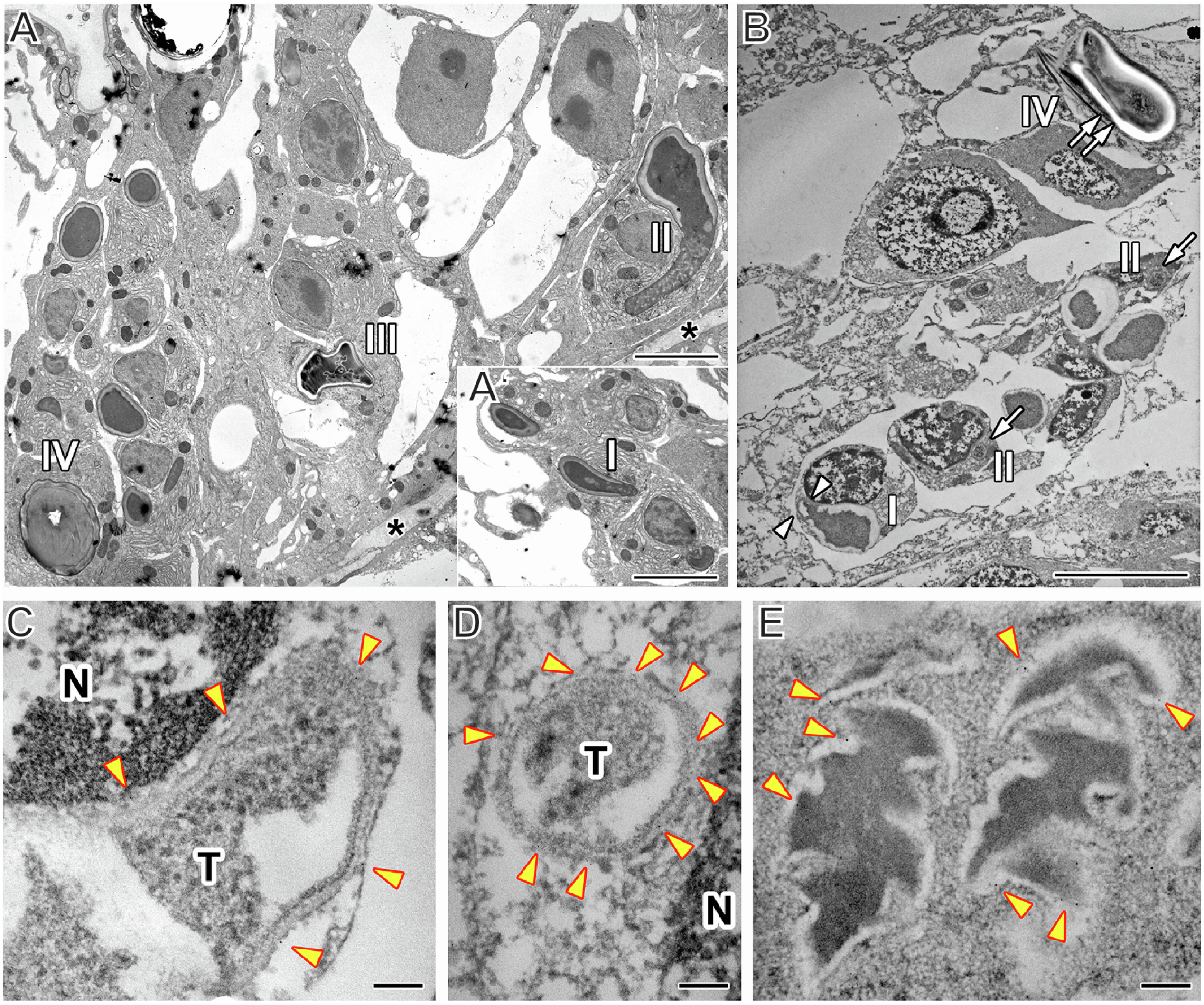

Fig. S1. Morphology of nematocyst development and localization of HyNMII by electron microscopy (EM), related to Fig. 1. (A) and (A') Overviews of cryofixed *Hydra* with all nematocyte developmental stages described here: I, onset of external tube formation (A'); II, tube elongation; III, maturing capsule with invaginated tube, IV mature capsule with fully coiled tube. Asterisks (*) marks the mesoglea. Scale bars = 0.5 µm. (B) Moderate, but acceptable ultrastructure preservation of nematocyte nests, as seen after sample fixation with MeOH + DMSO, tailored for HyNMII immuno-EM. Arrowheads highlight a stage I nematocyst with protruding tube, arrows point to cross-sectioned, stage II external tubes, double arrows to a stage III or IV nematocyte with inverted tube. Scale bar = 0.5 µm. (C), (D) and (E) HyNMII immuno-NANOGOLD™-silver particles, marked by arrowheads, locate at the cytoplasmic membrane face of the protruding (C) and elongating (D) tube, as well at the inverted tube (E). N = nucleus, T = tube. Scale bars = 200 nm.

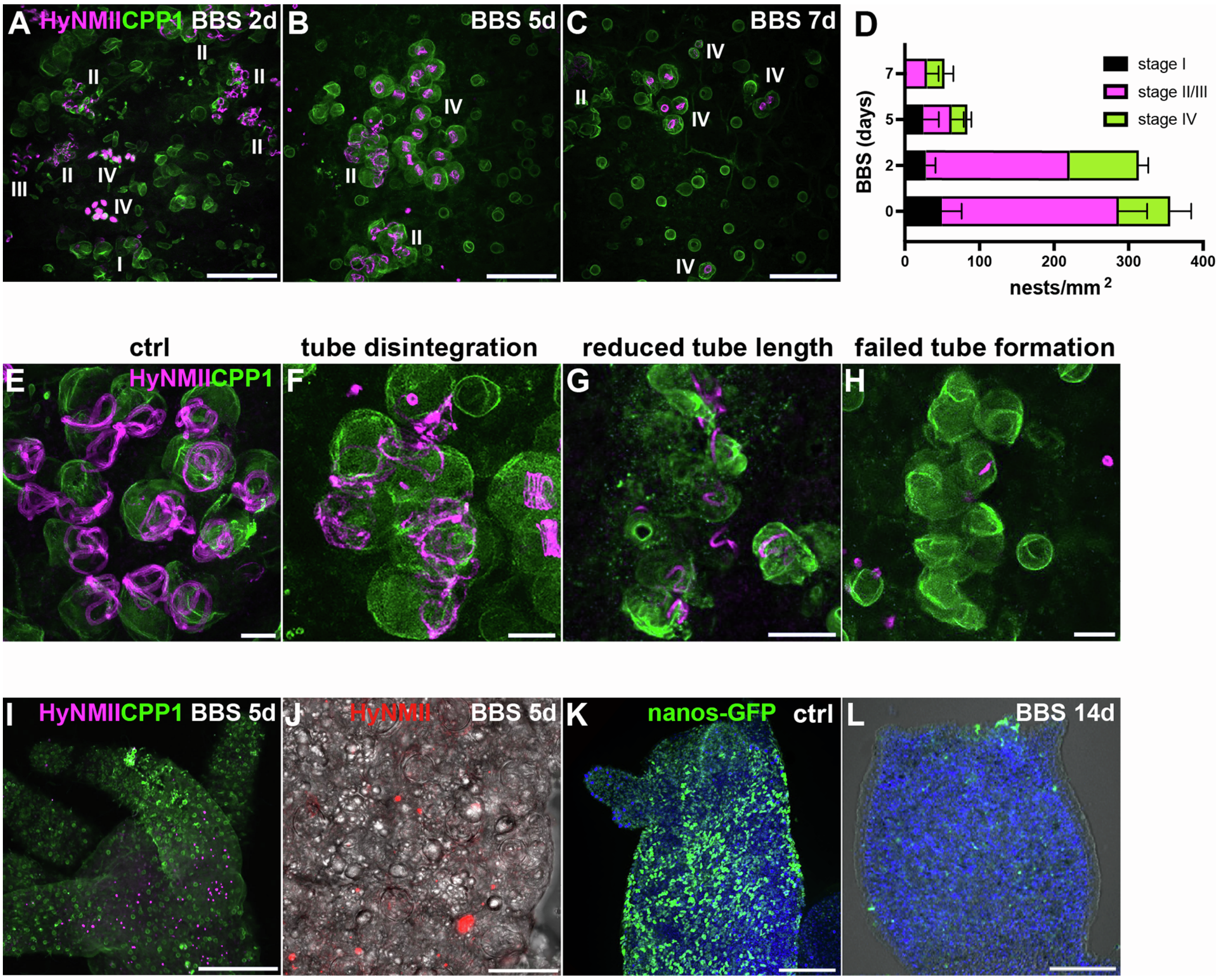

Fig. S2. Effect of Blebbistatin treatment on nematocyst morphogenesis and i-cell proliferation, related to Fig. 2. (A-C) Representative images of the gastric region in BBS treated polyps stained with HyNMII (magenta) and CPP1 (green) antibodies show a gradual loss of early developmental stages. While at day 2 of treatment (A) all stages are detectable, stage I-III nematocyst nests were dramatically depleted at days 5 and 7 (B-C), and at day 7 stage IV nests constituted nearly 43 % (19 % in controls) of the population while stage I nematocysts were not present anymore (C). This is confirmed by quantitative data shown in D. Note that stage IV nematocyst numbers were unaffected during the first 2 days of treatment. Stage II nematocysts (elongating external tube) at day 5 of BBS treatment (B) were associated with large HyNMII-positive particles indicating dissociation from the external tube as also shown in F. Scale bars = 50 µm. (D) Quantification of nematocyst stages in the gastric region of BBS-treated polyps at different days of treatment as in A-C (see also Table S5). Data represent mean +/− S.D. from at least 3 animals compared to control polyps. (E-H) Characteristic nematocyst phenotypes observed in BBS treated hydras as detected by HyNMII/CPP1 double staining. Most phenotypes affect tube morphogenesis at different stages of development. As compared to untreated polyps with fully developed external tubes (E), BBS treatment leads either to tube disintegration marked by HyNMII-positive puncta at tube tips (F), shortened tubes associated with nearly mature capsules (G) when affected at the tube elongation stage or completely failed tube formation in early stages as indicated by immature CPP1-positive capsule bodies lacking any HyNMII-positive tube structures (H). In rare cases, BBS treatment also leads to partial nematocyst capsule disintegration as in G. Scale bars = 10 µm. (I) Head region of a polyp showing HyNMII-positive dots in the tentacles. The animal was fixed at day 5 of BBS treatment and stained for HyNMII (magenta) and CPP1 (green). (J) Close-up image (differential interference contrast overlay) showing HyNMII-positive puncta (red) in battery cells of the tentacle. The animal was fixed at day 5 of BBS treatment. (K) Control animal of Cnnos1::GFP transgenic line is densely populated with GFP-positive i-cells in the gastric region. (L) Polyps continuously treated with BBS for 14 days are depleted of GFP-positive i-cells. Cell nuclei in J and K were stained with DAPI. Scale bars = I, K, L: 200 µm, J: 20 µm.

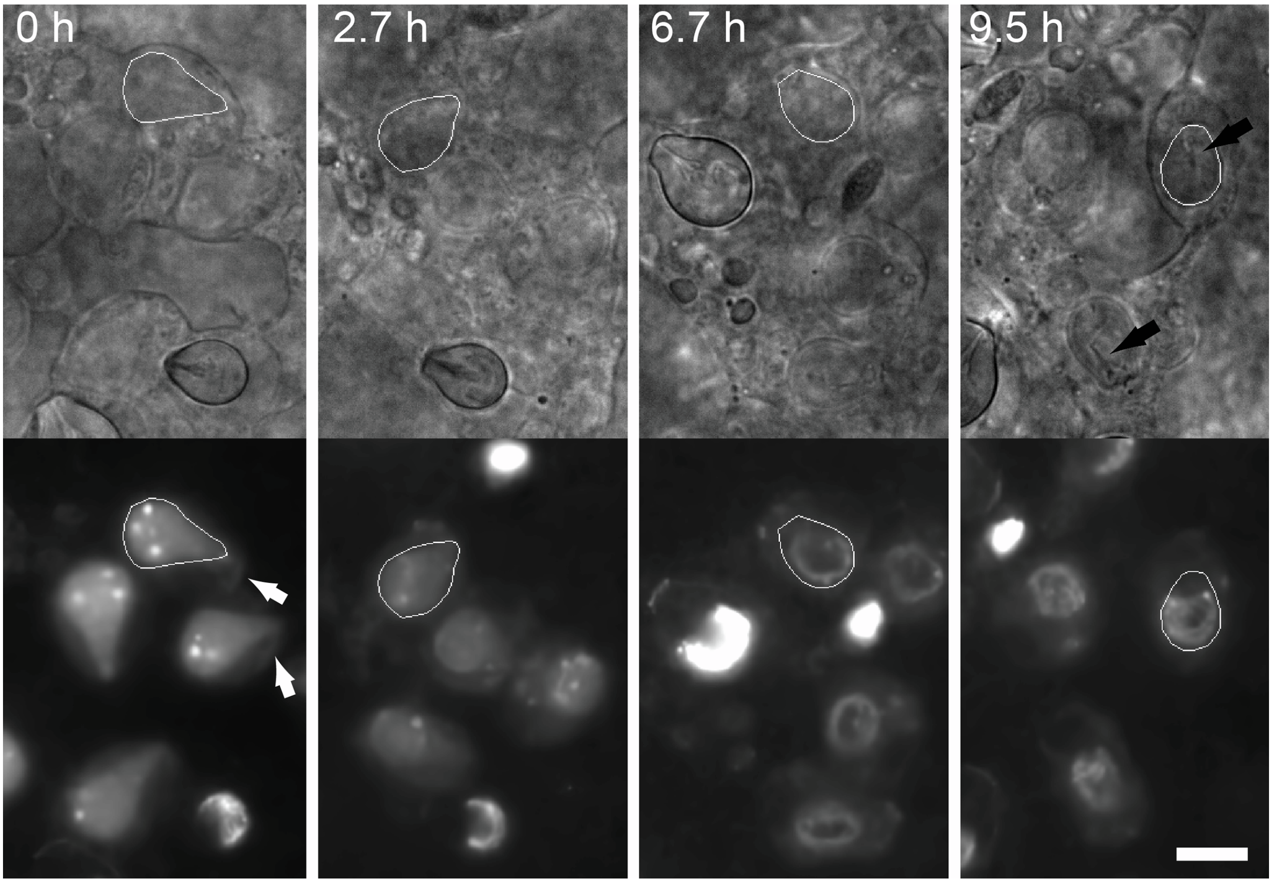

Fig. S3. Time lapse of NOWA-GFP and corresponding bright field images, related to Fig. 4 and Video S1. During the invagination process of the tube, NOWA aggregates continuously dissolve and NOWA protein associates with the tube. The contours of capsules were traced in the bright field images and overlayed on the fluorescence channel, the pointed end of the capsule indicates that the invagination is still ongoing, whereas after 6.7 hours invagination seems to be completed and the capsule has attained its final shape. White arrows point to external NOWA-GFP positive tube and black arrows highlight developing stylet apparatus at the end after invaginations is completed.

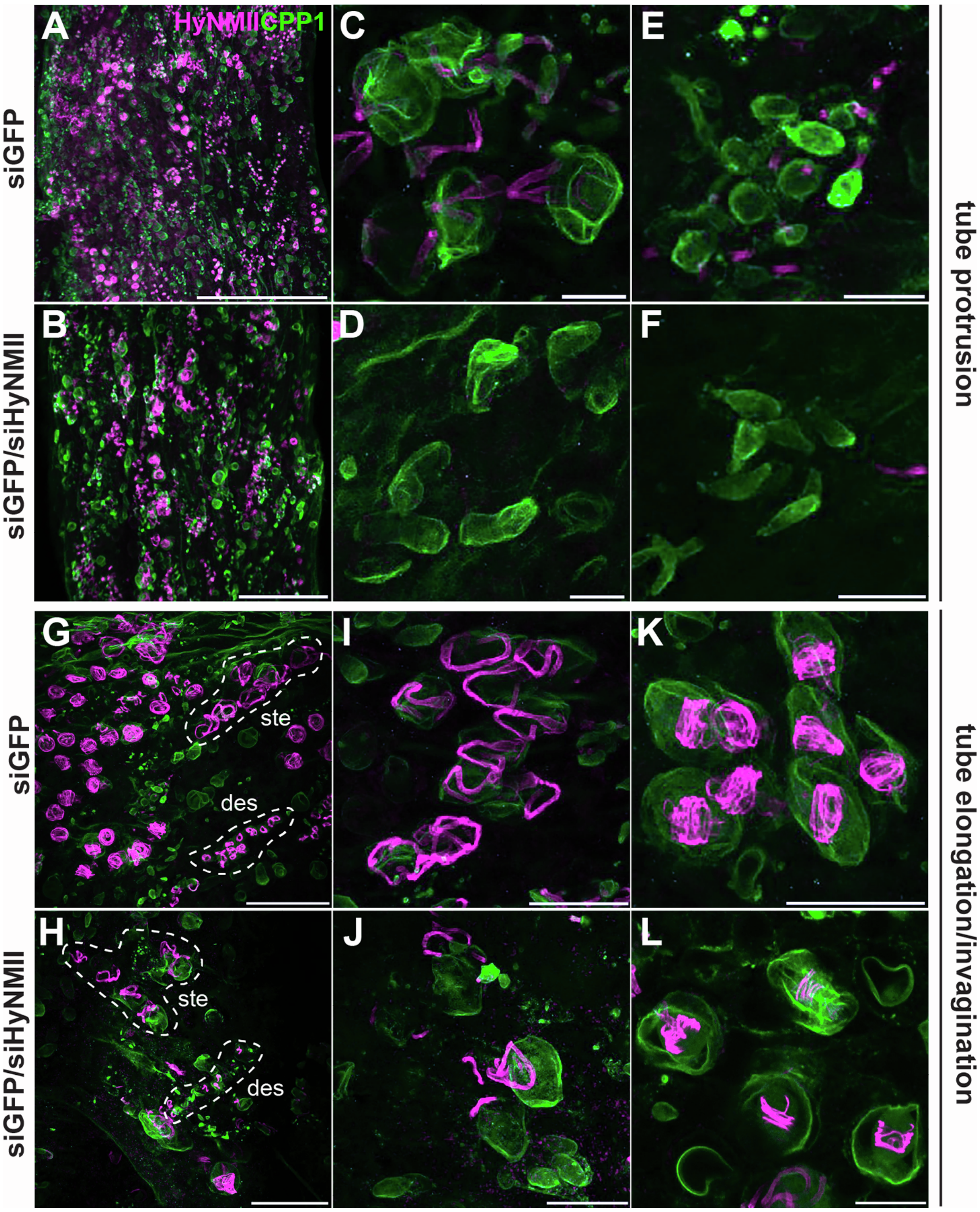

Fig. S4. Nematocyst phenotypes observed after *HyNMII* knockdown, related to Fig. 5. (A-B) Overview images of the gastric region co-stained with HyNMII (magenta) and CPP1 (green) antibodies show a reduction of the HyNMII signal in *GFP/HyNMII* siRNA electroporated animals (B) as compared to siGFP treated controls (A). HyNMII signal in tube protrusion stages (stage I) of stenoteles (C-D) and isorhizas (E-F) is shown to be completely lost. Overview images of the gastric region of siGFP (G) and siHyNMII/siGFP (H) electroporated animals show reduced tube lengths and loss of nematocysts in stage II (tube elongation) nests of stenoteles and desmonemes as indicated. (k-l) Close-up images of stage II (I-J) and stage IV (invaginated tube) stenotele nests show reduced tube lengths and partial loss of nematocysts in siGFP/siHyNMII treated animals compared to siGFP controls.

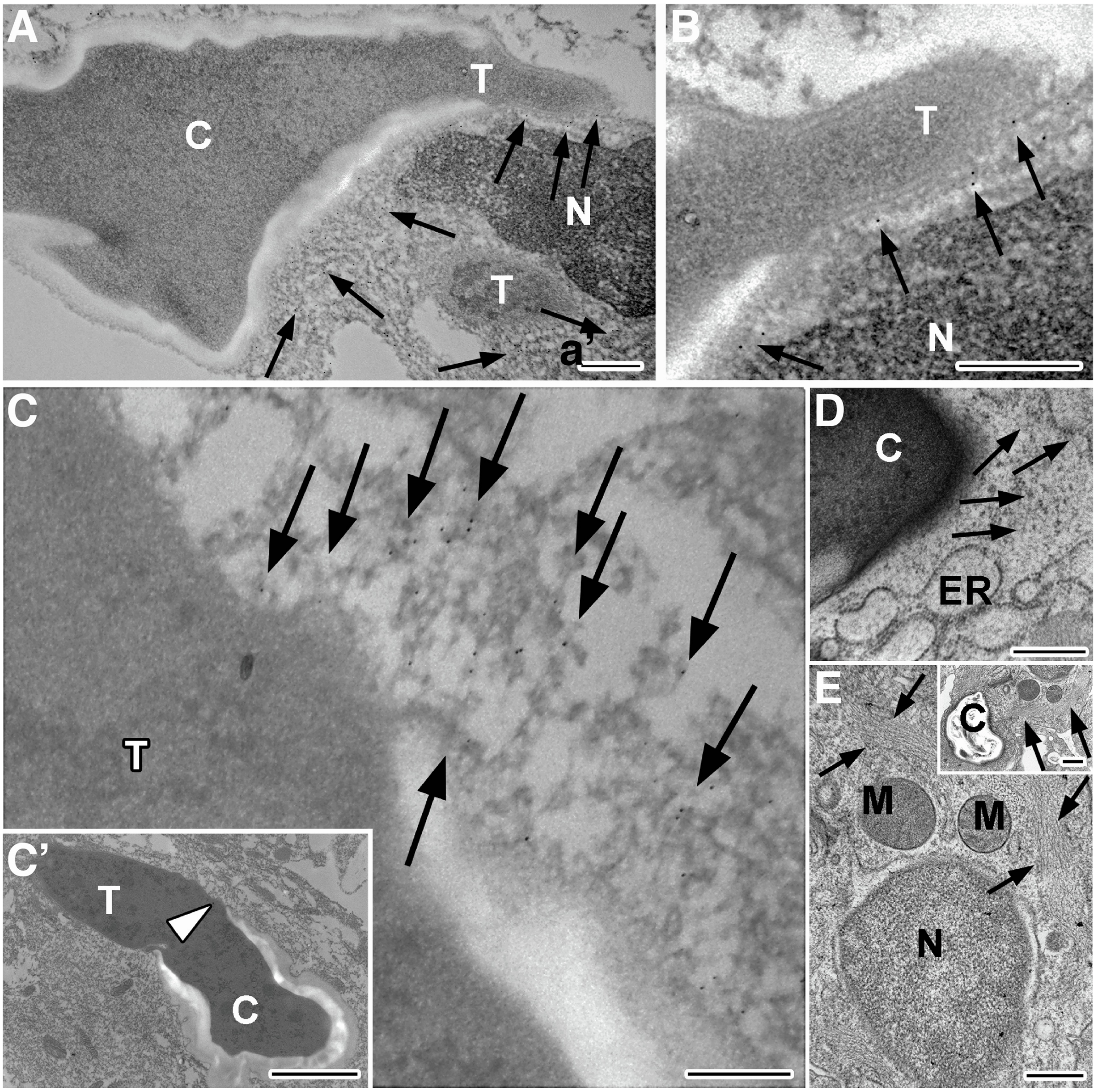

Fig. S5. Actin localization at the ultrastructural level, related to Fig. 6. (A) Actin, as visualized by immuno-NANOGOLD™-silver particles (arrows) in stage II nematocyte with elongating tube (T); N = nucleus, C = capsule. Formaldehyde + TX100 fixation. Scale bar = 500 nm. (B) Detail of Fig. S5A with label in the vicinity of the elongating tube (T). Scale bar = 500 nm. (C) Actin close to a stenotele's elongating tube. Arrows mark the orientation of fine filaments, being partly decorated by anti-actin immuno-NANOGOLD™-silver particles. Scale bar = 200 nm. Low magnification inset (C') shows the morphology of the respective stenotele with capsule (C) and tube (T); the arrow-head marks the area detailed in Fig. S5C. Scale bar = 2 µm. (D) Single 6-7 nm microfilaments (arrows) in close vicinity of the ER and tube protruding from the capsule (C) of cryofixed state I nematocyte. Scale bar = 500 nm. (E) Cryofixed Hydra sample showing bundles of approximately 7nm wide microfilaments (arrows) possibly representing actin, in stage III nematocyte with inverted tube; N = nucleus, M = mitochondria. Scale bar= 500 nm. Low magnification mirrored insert (E') of the respective nematocyte shows possible signs of capsule (C) disintegration. Scale bar = 500 nm.

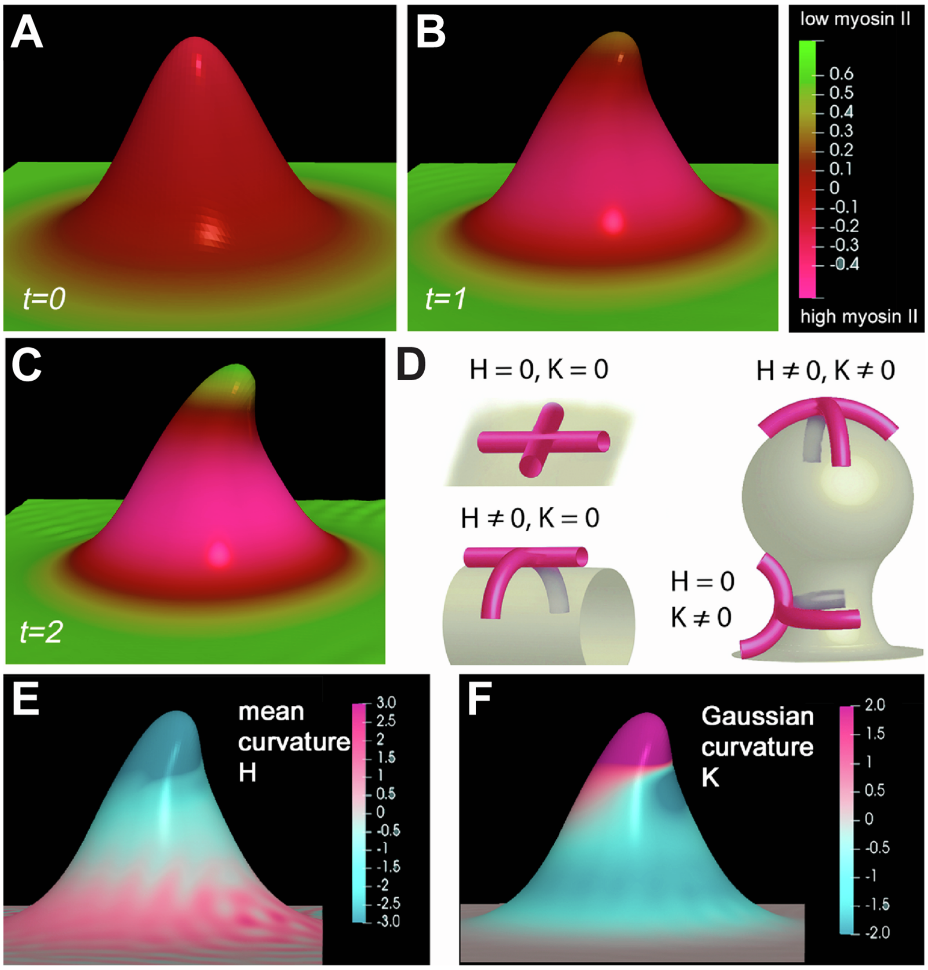

Fig. S6. Mathematical modelling and simulations of HyNMII-induced membrane shaping, related to Fig. 6. (A-C) simulation snapshots of evolving membrane geometry and lateral HyNMII distribution induced by a saddle-shaped protein structure. Magenta color corresponds to high HyNMII concentrations, green color to low concentrations. (D) Examples of simplified protein shapes represented by values of mean curvature H and Gaussian curvature K. (E) Mean curvature H, respectively (F) Gaussian curvature K for the membrane geometry shown in (D). Positive values are given in magenta color, negative values in blue color.

Table S1. Quantification of HyNMII-positive nests in the gastric region of BBS-treated polyps, related to Fig. 2F.

BBS treatment	Animal	Total number of HyNMII-positive nests	Area (μm)	Ratio of HyNMII-positive nests per area (μm)	Ratio of HyNMII-positive nests per area (mm^2)
Day 0	1	180	341113.42	0.00053	527.68
	2	266	689281.74	0.00039	385.91
	3	214	486759.09	0.00044	439.64
Day 1	1	85	274173.75	0.00031	310.02
	2	102	314898.77	0.00032	323.91
	3	144	380741.67	0.00038	378.21
Day 3	1	80	511791.54	0.00016	156.31
	2	58	537343.84	0.00011	107.94
	3	86	768748.81	0.00011	111.87
Day 5	1	37	412390.54	0.00009	89.72
	2	36	833245.21	0.00004	43.20
	3	54	977305.63	0.00006	55.25
Day 7	1	30	544114.81	0.00006	55.14
	2	24	499087.97	0.00005	48.09
	3	41	780409.61	0.00005	52.54

Table S2. Quantification of HyNMII-positive puncta, related to Fig. 2G.

Diameter of HyNMII-positive puncta (µm)			
3.34	2.44	2.49	3.97
2.76	3.46	1.80	2.45
2.46	1.57	3.26	1.62
1.70	3.24	3.03	1.15
3.18	1.44	3.55	4.61
3.87	1.48	2.52	3.25
2.62	2.43	3.59	3.44
2.64	2.99	2.05	1.97
1.87	3.36	1.90	1.66
2.61	1.62	1.55	1.90
2.12	2.22	3.05	4.04
1.54	1.70	2.87	2.27
2.23	2.12	3.64	1.64
2.10	2.57	3.52	1.56
3.40	1.72	2.46	3.21
2.47	3.80	2.87	4.60
2.33	2.05	3.09	3.95
2.20	2.81	3.89	1.99
2.03	2.49	3.54	2.23
2.79	2.22	2.84	2.99
2.88	2.02	3.80	2.89
3.12	2.44	3.50	3.81
2.17	3.46	2.75	3.79
2.96	1.57	2.71	1.90
2.12	3.24	2.41	3.14
2.64	1.44	3.01	3.18
2.64	1.48	3.73	3.03
1.73	2.43	2.19	2.58
3.38	2.99	3.26	3.97
1.65	3.36	3.23	2.45
2.01	1.62	3.13	1.62
3.23	2.22	3.09	1.15
3.17	1.70	3.45	4.61
3.55	2.12	3.25	3.25
2.54	2.57	2.46	3.44
1.85	1.72	3.88	1.97
1.88	3.80	4.44	1.66
3.49	2.05	2.75	1.90
2.85	2.81	1.80	4.04
4.12	2.49	1.65	2.27
2.71	2.22	2.21	1.64
3.20	2.02	2.08	1.56
3.07	3.29	2.26	3.21
3.47	2.01	2.15	4.60

Table S3. Quantification of Ncol1-pp-positive nests, related to Fig. 3M.

BBS treatment	Animal	Total number of stenotele nests	Area (μm)	Ncol1-pp-positive nests per area (mm^2)
**Day 0**	1	73	1321822.08	55.23
	2	86	2042550.84	42.10
	3	38	508854.43	74.68
**Day 1**	1	13	445696.30	29.17
	2	34	979968.73	34.69
	3	52	1490796.02	34.88
**Day 2**	1	16	345732.33	46.28
	2	10	255562.20	39.13
	3	25	868696.95	28.78
	4	39	1156989.60	33.71
**Day 3**	1	14	952799.33	14.69
	2	49	2038209.60	24.04
	3	30	917068.59	32.71
**Day 4**	1	14	443825.72	31.54
	2	18	545148.57	33.02
	3	56	2008104.27	27.89
**Day 5**	1	6	342225.82	17.53
	2	29	1351515.92	21.46
	3	15	880838.30	17.03
**Day 6**	1	3	570398.47	5.26
	2	7	851849.65	8.22
	3	6	392561.04	15.28
**Day 7**	1	6	695192.56	8.63
	2	7	1275966.05	5.49
	3	12	1415474.10	8.48

Table S4. Quantification of partially invaginated nests, related to Fig. 4L.

	Closeup	Partially invaginated nests	Other stages	Total number of nests	Partially invaginated nests (%)	Average partially invaginated nests (%)
**Control (3 Animals)**	1	0.00	16.00	16.00	0.00	5.22
	2	0.00	20.00	20.00	0.00	
	3	5.00	19.00	24.00	20.83	
	4	1.00	17.00	18.00	5.56	
	5	3.00	11.00	14.00	21.43	
	6	2.00	19.00	21.00	9.52	
	7	0.00	14.00	14.00	0.00	
	8	0.00	16.00	16.00	0.00	
	9	1.00	15.00	16.00	6.25	
	10	3.00	15.00	18.00	16.67	
	11	1.00	11.00	12.00	8.33	
	12	0.00	16.00	16.00	0.00	
	13	1.00	24.00	25.00	4.00	
	14	1.00	15.00	16.00	6.25	
	15	1.00	17.00	18.00	5.56	
	16	0.00	19.00	19.00	0.00	
	17	0.00	12.00	12.00	0.00	
	18	0.00	13.00	13.00	0.00	
	19	0.00	16.00	16.00	0.00	
	20	0.00	18.00	18.00	0.00	
**siNOWA (3 animals)**	1	3.00	13.00	16.00	18.75	17.96
	2	5.00	20.00	25.00	20.00	
	3	3.00	24.00	27.00	11.11	
	4	4.00	12.00	16.00	25.00	
	5	2.00	14.00	16.00	12.50	
	6	2.00	18.00	20.00	10.00	
	7	1.00	14.00	15.00	6.67	
	8	2.00	16.00	18.00	11.11	
	9	4.00	13.00	17.00	23.53	
	10	2.00	8.00	10.00	20.00	
	11	2.00	16.00	18.00	11.11	
	12	3.00	19.00	22.00	13.64	
	13	4.00	15.00	19.00	21.05	
	14	4.00	18.00	22.00	18.18	
	15	4.00	23.00	27.00	14.81	
	16	2.00	18.00	20.00	10.00	
	17	2.00	17.00	19.00	10.53	
	18	5.00	14.00	19.00	26.32	
	19	4.00	11.00	15.00	26.67	
	20	2.00	14.00	16.00	12.50	
	21	9.00	20.00	29.00	31.03	
	22	8.00	10.00	18.00	44.44	
	23	9.00	11.00	20.00	45.00	
	24	5.00	9.00	14.00	35.71	
	25	3.00	17.00	20.00	15.00	
	26	2.00	17.00	19.00	10.53	
	27	6.00	19.00	25.00	24.00	
	28	2.00	20.00	22.00	9.09	
	29	3.00	18.00	21.00	14.29	
	30	5.00	25.00	30.00	16.67	
	31	2.00	19.00	21.00	9.52	
	32	2.00	20.00	22.00	9.09	
	33	3.00	14.00	17.00	17.65	
	34	1.00	19.00	20.00	5.00	

Table S5. Quantification of HyNMII-positive nest types, related to Fig. 2 and Fig. S2D.

Stage I (nests/mm^2)				
**Day 0**	**Day 2**	**Day 5**	**Day 7**	
42.75	42.84	42.84	0	
21.38	21.42	64.26	0	
42.75	21.42	42.84	0	
85.5		21.38	0	
64.13		0		
0		21.38		
64.13		21.38		
64.13		42.75		
64.13		0		
49.88	28.56	25.17	0	**Mean**
26.18	12.37	20.35	0	**STD**

Stage II/III (nests/mm^2)				
235.14	192.79	42.84	63.98	
213.76	192.79	42.84	0	
299.26	192.79	42.84	64.13	
256.51		21.42	42.75	
213.76		21.42		
171.01		64.13		
277.89		64.13		
235.14		21.38		
235.14		21.38		
		21.38		
		21.38		
		21.38		
		42.75		
		42.75		
		42.75		
		42.75		
		64.13		
237.5	192.8	37.74	30.2	**Mean**
37.7	0	16.08	15.1	**STD**

Stage IV (nests/mm^2)				
0	85.68	21.42	0	
85.5	85.68	42.84	0	
64.13	107.1	0	49.06	
85.5		0	21.38	
64.13		21.38		
64.13		21.38		
85.5		0		
85.5		42.75		
85.5		0		
		0		
		21.38		
		42.75		
		0		
		0		
		0		
		64.13		
		42.75		
68.88	92.82	21.22	23.26	**Mean**
27.82	12.37	5.148	11.63	**STD**
